# The cardioprotective effect of dexrazoxane (Cardioxane) is consistent with sequestration of poly(ADP-ribose) by self-assembly and not depletion of topoisomerase 2B

**DOI:** 10.3332/ecancer.2018.889

**Published:** 2018-12-20

**Authors:** Keith McCormack

**Affiliations:** McCormack Pharma, a division of McCormack Ltd, Stirling House, 9 Burroughs Gardens, London NW4 4AU, UK

**Keywords:** dexrazoxane, anthracyclines, cardioprotection, poly(ADP-ribose), topoisomerase 2β, epigenome, Watson Crick

## Abstract

Following systematic scrutiny of the evidence in support of the hypothesis that the cardioprotective mechanism of action of dexrazoxane is mediated by a ‘depletion’ or ‘downregulation’ of Top2β protein levels in heart tissue, the author concludes that this hypothesis is untenable. In seeking to understand how dexrazoxane protects the heart, the outcomes of a customised association rule learning algorithm incorporating the use of antecedent surrogate variables (CEME, 2017 McCormack Pharma) reveal a previously unknown relationship between dexrazoxane and poly(ADP-ribose) (PAR) polymer. The author shows how this previously unknown relationship explains both acute and long-term cardioprotection in patients receiving anthracyclines. In addition, as a direct inhibitor of PAR dexrazoxane has access to the epigenome and this offers a new insight into protection by dexrazoxane against doxorubicin-induced late-onset damage [McCormack K, manuscript in preparation]. Notably, through this review article, the author illustrates the practical application of probing natural language text using an association rule learning algorithm for the discovery of new and interesting associations that, otherwise, would remain lost. Historically, the use of CEME enabled the first report of the capacity of a small molecule to catalyse the hybrid self-assembly of a nucleic acid biopolymer via canonical and non-canonical, non-covalent interactions analogous to Watson Crick and Hoogsteen base pairing, respectively.

## The acute cardioprotective effect of dexrazoxane

## Introduction

### A brief historical perspective

**Figure 1.**
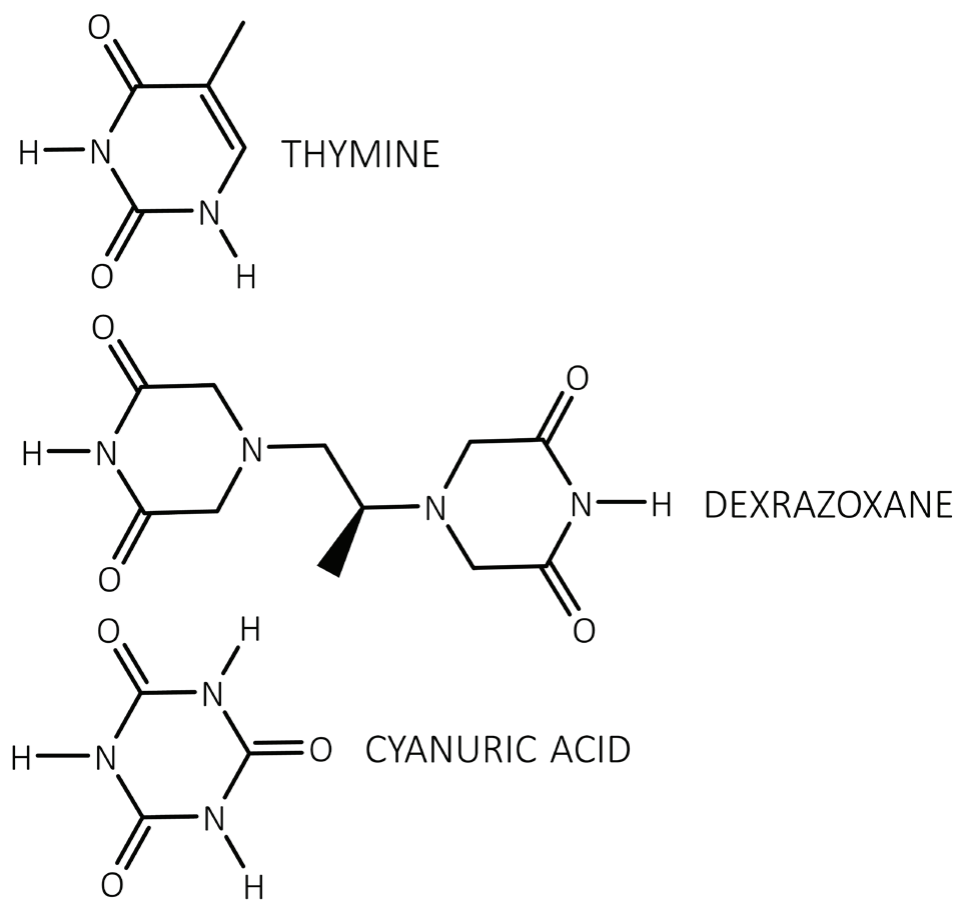
Each dioxopiperazine moiety of dexrazoxane contains a thymine face and cyanuric acid has three thymine faces.

Dexrazoxane (ICRF-187) (Figure 1) belongs to the bisdioxopiperazine class of compounds and is a water-soluble ring-closed analogue of the iron chelator ethylenediaminetetraacetic acid (EDTA). Upon hydrolysis, dexrazoxane opens into its EDTA-like form, ADR-925, which is a strong iron chelator with the ability to displace iron bound to an anthracycline [169].

The interest in the bisdioxopiperazines as potential protectors against anthracycline-mediated myocardial damage emerged from a large series of preclinical studies, notably those performed by Herman* et al* throughout the 1970s and 1980s [170]. Pre-treatment with dexrazoxane reduced the cardiotoxicity and lethality in non-cancer-bearing Syrian golden hamsters receiving daunorubicin [171]. Furthermore, pre-treatment with dexrazoxane was shown to be cardioprotective in doxorubicin- and daunorubicin-treated miniature swine, beagle dogs and rabbits [172–174]. Later, clinical studies in humans confirmed the cardioprotection exerted by dexrazoxane.

Historically, many mechanisms have been proposed to explain the molecular basis of anthracycline-induced cardiotoxicity. However, the most-investigated mechanism describes the ability of this class of drugs to disrupt iron metabolism and generate an excess of reactive oxygen species (ROS) within cardiomyocytes, with DNA damage resulting from increased oxidative stress and lipid peroxidation [175–178]. From a molecular perspective, the chemical structure of doxorubicin is particularly prone to the generation of free radicals as doxorubicin can be reversibly reduced to a semiquinone, an unstable metabolite whose futile cycling within mitochondria releases ROS [179]. More generally, as a class-effect anthracycline-dependent oxidative stress has long been ascribed to the ability of these drugs to chelate free iron, thereby forming iron–anthracycline complexes which, in turn, react with oxygen and trigger ROS production [180, 181]. Overall, numerous studies emphasise the critical role of free iron in anthracycline-induced cardiotoxicity and suggest that reduction of iron levels may constitute an effective strategy to prevent anthracycline-induced heart failure.

However, numerous antioxidants and prototypic ROS scavengers that include N-acetylcysteine, coenzyme Q, vitamins C and E, and several iron chelators have been tested *in vitro* and *in vivo* with variable outcomes [64]. None of these compounds have matched or even surpassed the effectiveness of dexrazoxane in chronic anthracycline cardiotoxicity settings, despite being stronger chelators or antioxidants.

By way of illustration, deferiprone is an orally effective α-ketohydroxypyridine iron chelator. As a small lipophilic molecule, deferiprone penetrates readily into various tissues and has been shown to remove excess iron from the heart [182] and bind labile free and accumulated cellular iron within mitochondria and lysosomes [183]. Nevertheless, using a clinically relevant and dexrazoxane-validated model of chronic anthracycline-induced cardiotoxicity in rabbits [184], deferiprone showed no potential to alleviate either anthracycline-induced oxidative stress or left ventricular cardiac damage and congestive heart failure, as assessed by both echocardiography and left ventricular catheterisation [185]. Deferasirox, a clinically approved iron chelator likewise failed to protect isolated rat cardiomyocytes against doxorubicin-induced damage despite the observations that this drug rapidly entered cardiomyocytes and efficiently removed iron from its complex with doxorubicin [186].

In summary, studies with various iron-specific chelators (both clinically approved drugs as well as experimental agents) have yielded rather mixed results, particularly when evaluated using clinically relevant animal models of anthracycline-induced cardiotoxicity. Notably, none of these agents achieved the model-independent protective efficiency of dexrazoxane even though the studied agents are even stronger and more selective intracellular iron chelators than dexrazoxane.

Vavrova* et al* [64] investigated the involvement of Top2 in anthracycline-induced cardiotoxicity. Using primary cultures of isolated rat neonatal ventricular cardiomyocytes, they examined the protective effects of dexrazoxane against cardiotoxicity induced by daunorubicin and doxorubicin. From the outcomes of this meticulous and systematic investigation, these workers conclude that, in agreement with previous suggestions, the cardioprotective effect of dexrazoxane is not adequately explained by iron chelation or antioxidant properties. Rather, in consideration of reports of the role of Top2β in mediating anthracycline-mediated cardiotoxicity [1–3], notably that by Zhang* et al* [1] and Lyu* et al* [4], they propose that the selective degradation of Top2β is a plausible explanation for the cardioprotective effects of dexrazoxane.

### Top2β is the molecular target of doxorubicin

Anthracyclines, notably doxorubicin are widely used chemotherapeutic agents. However, and as briefly discussed above, their clinical use is often limited by dose-dependent cardiotoxicity. Zhang* et al* hypothesised that doxorubicin-induced cardiotoxicity is mediated by Top2β, the only Top2 enzyme expressed in cardiomyocytes [1]. These workers elegantly show that cardiomyocyte-specific deletion of *Top2β* (encoding Top2β) protects cardiomyocytes from doxorubicin-induced DNA double-strand breaks (DSBs) and transcriptome changes that are responsible for defective mitochondrial biogenesis and the generation of ROS. Importantly, they also show that cardiomyocyte-specific deletion of *Top2β* protects mice from the development of doxorubicin-induced progressive heart failure, providing robust support for the view that doxorubicin-induced cardiotoxicity is mediated by Top2β in cardiomyocytes. They conclude that the molecular phenotype of acute and chronic doxorubicin cardiomyopathy is characterised by the formation of a ternary DNA–Top2β–doxorubicin cleavage complex, that triggers DSBs in DNA, in part by preventing religation of Top2β-induced strand breaks.

In accord with these erudite observations, Velpongsa and Yeh [2] propose that inhibiting and deleting Top2β in the heart should be tested as a strategy for the primary prevention of anthracycline-induced cardiotoxicity, and in a recent review, Moudgil and Yeh [3] conclude that Top2β is required to initiate the entire phenotypic cascade of doxorubicin-induced cardiomyopathy.

### Dexrazoxane induces degradation of Top2β

Several investigators have observed depletion of the doxorubicin target Top2β in cells treated with dexrazoxane. From their results using H9C2 cardiomyocytes, Lyu* et al* [4] suggest that dexrazoxane may prevent doxorubicin-induced DNA damage by triggering proteasomal degradation of Top2β. They argue that dexrazoxane binds to theTop2β-DNA binary complex and stabilises Top2β as a closed clamp upon DNA. This clamp blocks the movement of the transcription-elongation complex resulting in transcriptional arrest, which, in turn, triggers 26S proteasome-dependent degradation of Top2β, a well-documented mechanism for clearing Top2-mediated ‘transcription-road blocks’ [6–8].

Deng* et al* [42] report depletion of Top2β protein in the hearts of C57BL (B6) mice following the intraperitoneal administration of dexrazoxane. Together with the results of additional systematic and complementary *in vitro* investigations, these workers describe a model in which dexrazoxane depletes cardiac Top2β, thereby reducing the substrate for ‘doxorubicin poisoning’. Accordingly, subsequent administration of doxorubicin is anticipated to effect lower levels of DNA damage by comparison with no dexrazoxane pre-treatment.

A multidisciplinary collaborative effort between the Faculties of Pharmacology, Pharmacy and Medicine at Charles University in Hradec Králové, Czech Republic [162–164], was undertaken to investigate the molecular basis for the cardioprotective effect of dexrazoxane using rabbits together with H9C2 cells and freshly harvested ventricular rat cardiomyocytes. From the results of this conjoint study, the workers report that in rabbits, pre-treatment with dexrazoxane completely prevents daunorubicin-induced heart damage. Notably, this protection was unrelated to an effect upon oxidation status and dexrazoxane was shown to exert no effect upon mobilisation of intracellular iron. However, experiments both *in vivo* and *in vitro* showed a decrease in Top2β protein levels within rabbit heart and cell cultures following exposure to dexrazoxane. Taken together, they conclude that their findings support the view that the cardioprotective effects of dexrazoxane may not be attributable to iron chelation and/or mitigation of oxidative stress; rather, they suggest that depletion of Top2β may represent the molecular basis for the cardioprotective action of dexrazoxane.

In a series of National Institute of Health funding applications for the fiscal years 2015, 2016, 2017 and 2018, Yeh provides details of his proposed research aims that include a determination of whether dexrazoxane-induced degradation of Top2β in the heart prevents doxorubicin-induced cardiotoxicity, and by what mechanism does dexrazoxane induce degradation of Top2β [9, 165–168]. Within the formal abstracts of these applications, Yeh alludes to preliminary studies in which he and his coworkers showed that dexrazoxane induced degradation of Top2β through a proteasome-dependent mechanism. Indeed, the outcomes of studies detailed within the earlier Patent Application US 14/155,858 are presented by the same worker in support of the claim therein that the cardioprotective compound dexrazoxane functions in some part by reducing Top2β protein levels in heart tissue [5]. Interestingly, within that application, the inventor showed that dexrazoxane-mediated Top2β degradation was inhibited by the proteasome inhibitor MG132 suggesting a proteasome-dependent mechanism for the effects of dexrazoxane upon the level of Top2β protein expression.

Collectively, the outcomes presented above are seductive and in part have given rise to the burgeoning hypothesis, evident within an abundant scientific and clinical literature that dexrazoxane protects the heart against anthracycline-induced damage by promoting the proteasomal degradation of Top2β, the Top2 isoenzyme that predominates in cardiomyocytes.

### Does the cardioprotective mechanism of dexrazoxane involve Top2β?

In summary, the evidence in support of a pivotal role of Top2β in mediating doxorubicin-induced cardiotoxicity and heart failure is robust and convincing.

However, as this systematic analysis shows the presumed corollary that the cardioprotective mechanism of action of dexrazoxane is mediated by a ‘depletion’ or ‘downregulation’ of Top2β protein levels in heart tissue is untenable and incorrect. Indeed, classically, this ‘new hypothesis’ is a category error that contains the fallacy of division (assumes that the part has the properties of the whole).

If dexrazoxane protects the heart against doxorubicin-induced damage by suppressing Top2β protein levels in heart tissue, then two criteria must be satisfied absolutely and unequivocally:

- Following a single infusion of dexrazoxane, Top2β protein levels in heart tissue must be suppressed to, or below some threshold whereby Top2β protein levels no longer represent a molecular target for the effects of doxorubicin.
- Dexrazoxane-suppressed Top2β protein levels in heart tissue must remain at, or below the above threshold during the period that the heart is exposed to damaging levels of doxorubicin.

In seeking to establish whether these criteria are satisfied, scrutiny of the available literature is necessary to establish the period during which the levels of doxorubicin within heart tissue can be reasonably expected to be toxic following single-dose administration. Within this window of cardiotoxicity, it remains to determine whether a single dose of dexrazoxane administered prior to doxorubicin can suppress levels of Top2β protein levels in heart tissue to or below a threshold at which they do not represent a molecular target for doxorubicin for the entire period during which doxorubicin residence poses a threat to cardiomyocyte viability.

## Accumulation of doxorubicin within the heart

### Study duration 24 hours

Using Friend Leukaemia Virus B (FVB) mice, van Asperen’s group report mean doxorubicin concentrations of 21.5, 12.6 and 1.7 μmol kg^−1^ in the hearts of mice sacrificed at 1, 4 and 24 hours, respectively, following the administration of doxorubicin into the tail vein at a dose of 5 mg kg^−1^ [10]. Nwankwoala* et al* studied the pharmacokinetics and tissue distribution of doxorubicin in Wistar rats. They report mean doxorubicin concentrations of 72.4, 47.8, 26.7 and 13.0 μmol kg^−1^ in the hearts of rats sacrificed at 1, 3, 12 and 24 hours, respectively, following the intravenous administration of doxorubicin at a dose of 20 mg kg^−1^. Administering doxorubicin at a dose of 1 mg kg^−1^ they report mean doxorubicin concentrations of 6.7, 2.8 and 1.5 μmol kg^−1^ in the hearts of rats sacrificed at 1, 12 and 24 hours, respectively [11].

Using Sprague-Dawley rats, Rahman* et al* [12] compared the pharmacokinetic and tissue disposition of doxorubicin with that of doxorubicin encapsulated within cardiolipin-modified liposomes. They report mean doxorubicin concentrations of 27.8, 21.2, 18.03 and 8.6 μmol kg^−1^ in the hearts of rats sacrificed at 30 minutes, 2, 4 and 24 hours, respectively, following the administration of free doxorubicin into the femoral vein at a dose of 6 mg kg^−1^. At the same time, they report mean cardiac concentrations of 16.2, 16.0, 10.7 and 4.2 μmol kg^−1^, respectively, following the administration of the liposomal-formulated doxorubicin at a dose of 6 mg (doxorubicin) kg^−1^ [12].

### Study duration 48 hours

Ozols* et al* [13] studied the tissue disposition of doxorubicin in C3HeB/FeJ mice with a transplantable ovarian cancer. They report mean doxorubicin concentrations of 18.4, 7.4 and 1.2 μmol kg^−1^ in the hearts of mice that were sacrificed at 15 minutes, 24 and 48 hours, respectively, following the administration of doxorubicin at an LD10 (lethal dose to 10% of animals) dose of 10 mg kg^−1^ into the tail vein (numerical data extracted by digitisation of graphical plots).

More recently, Staples* et al* [14] studied the distribution of doxorubicin following the use of low-frequency ultrasound to trigger the release of micelle-encapsulated doxorubicin in Berlin Druckrey strain IX (BDIX) rats with bilateral leg tumours following injection of the DHD/K12/TRb colorectal cell line. They report mean doxorubicin concentrations of 10.4, 4.7, 4.4, 3.05, 1.6 and 0.8 μmol kg^−1^ in the hearts of rats that were sacrificed at 30 minutes, 6, 8, 12, 24 and 48 hours, respectively, following the administration of encapsulated-doxorubicin into the lateral tail vein at a dose of 2.67 mg (doxorubicin) kg^−1^.

### Study duration beyond 48 hours

Staples* et al* [14] additionally report detectable levels of doxorubicin in the hearts of rats that were sacrificed at 96 and 168 hours. Interestingly, in a subgroup of rats, repeated weekly-administrations of encapsulated doxorubicin over a 4-week period resulted in a significantly greater amount of doxorubicin in the heart by comparison with that following a single treatment (*p* = 0.044), with no statistically significant differences in concentrations between the single and multiple treatment groups in the liver, leg muscle and tumour tissues (*p* = 0.262, *p* = 0.397 and *p* = 0.327, respectively) (numerical data extracted by digitisation of graphical plots). These findings support the view (see below) that the heart selectively accumulates doxorubicin.

Consistent with the report by Staples* et al* [14] of detectable levels of doxorubicin at long time points, Chenard [15] reported detectable levels of doxorubicin at 192 hours in the hearts of Sprague Dawley rats following the intraperitoneal administration of doxorubicin at a dose of either 1.5 or 4.5 mg kg^−1^.

### Long-term retention of doxorubicin within the heart (animal data)

The persistence of doxorubicin within myocardial cells had been previously demonstrated by the early histofluorescence study by Amin* et al* [16]. Following the intravenous administration of doxorubicin at a dose of 15 mg kg^−1^ to Sprague Dawley rats, fluorescence emission was observed in myocardial cells of the left ventricle until 28 days after administration.

### Long-term retention of doxorubicin within the heart (patient data)

In accord with the outcomes from animal studies, Stewart* et al* [17] demonstrated the prolonged retention of doxorubicin within autopsy cardiac tissue from 35 patients who had received doxorubicin at any time antemortem. The median lifetime cumulative doxorubicin dose was 268 mg m^−2^ (range 30–670 mg m^−2^), and the median time from the last doxorubicin treatment to death was 81 days (range, 1–931 days). The median doxorubicin concentration in cardiac tissue was 0.11 μmol kg^−1^ (range, 0–3.06 μmol kg^−1^). These workers add that their results suggest that cardiomyocytes selectively accumulate doxorubicin by comparison with other types of muscle.

### Doxorubicin displays rapid uptake and slow elimination

Taken together, the data presented above are consistent with a prolonged terminal half-life of doxorubicin and a propensity to accumulate within cardiac tissue. In humans, free doxorubicin has a distributive half-life of about 5 minutes and a terminal half-life of 20–48 hours, indicating fast uptake into tissues and slow elimination thereafter [18]. High-affinity binding of doxorubicin with the diphosphatidylglycerol lipid, cardiolipin located within the inner mitochondrial membrane, may explain in some part the retention of doxorubicin by the mitochondrion-rich cardiomyocytes [19].

### Can we determine a cardiotoxic limiting concentration of doxorubicin?

While the studies reviewed above show measurable concentrations of doxorubicin at extended time points in the hearts of both animals and humans, how can we identify a limiting concentration, below which doxorubicin is not overtly cardiotoxic?

The half-maximal inhibitory concentration (IC50) of doxorubicin as a measure of potency in reducing the viability of cardiomyocytes provides a useful datum for enabling perspective. The author identified ten studies that report IC50 values for growth inhibition of cardiomyocytes using either free or liposomal-encapsulated doxorubicin, with a median value of 1.67 μM: 0.02, 0.04, 0.12, 0.31, 1.33, 2.0, 3.09, 3.5, 3.6 and 5.6, with units as μM [20–28].

### Accumulation of doxorubicin suggests long-term effects on Top2β

Accepting the proportionality error in comparing concentrations in an aqueous buffer (IC50, μmol dm^−3^ = μM) with concentrations in *ex vivo* tissue mass (μmol kg^−1^), then using the literature values of IC50 as a measure of toxicity, it can be concluded that for prolonged periods the cardiac concentrations of doxorubicin are sufficient to negatively impact upon cardiomyocyte viability. The limited animal data suggest that cardiotoxic levels of doxorubicin remain significantly above the median IC50 value of 1.67 μM (see above) for at least 24 hours and decline below this limiting concentration between 24 and 48 hours. In both animals and patients, there is convincing evidence for long-term (days/weeks) retention of doxorubicin within cardiac tissue. Accordingly, given the long-term accumulation of doxorubicin within the heart, if the short pre-administration single infusion of dexrazoxane exerts a cardioprotective effect by depleting Top2β protein, then by necessity suppression of the level of Top2β protein within the heart must be profound and long lasting. As the author argues, this seems a remote prospect given that by comparison with the slow elimination of doxorubicin, dexrazoxane has a remarkably short half-life. The logical corollary of this substantial difference in pharmacokinetics is twofold. First, dexrazoxane must exert long-lasting suppression that results in maintenance of Top2β protein below threshold. Second, in the absence of long-lasting suppression, recovery of suppressed Top2β protein (transcription) must be slow.

## Does dexrazoxane eliminate cardiac Top2β as a target?

If dexrazoxane protects the heart against doxorubicin-induced damage by suppressing Top2β protein levels, then Top2β protein levels in heart tissue must be suppressed to, or below some threshold whereby Top2β protein levels no longer represent a molecular target for the effects of doxorubicin. As a prelude to reviewing the literature reports that describe the suppression of cardiac Top2β protein levels by dexrazoxane, detailed attention must be given to the pharmacokinetics of both dexrazoxane and doxorubicin since the duration of any suppressive effects will be determined by the residence time of dexrazoxane and the natural turnover of Top2β protein.

### Dexrazoxane is slowly metabolised in vitro

Under physiological conditions (37°C and pH 7.4) *in vitro*, dexrazoxane is slowly hydrolysed to the single-ring-opened metabolites (described elsewhere as B and C) (*t*_1/2_ of 9.3 hours), and to the final hydrolysis product ADR-925 (*t*_1/2_ of 23 hours) [29–32]. Given the slow rate at which dexrazoxane hydrolysis occurs *in vitro*, considerable caution must be exercised in extrapolating laboratory findings using cell preparations exposed continuously to high concentrations of dexrazoxane, to the clinical setting with rapidly declining concentrations of dexrazoxane.

### In a clinical setting, the metabolism of dexrazoxane is extremely rapid

Schroeder* et al* [33] investigated the metabolism of dexrazoxane in cancer patients with brain metastases treated with high-dose etoposide. They showed that the two single-ring-opened hydrolysis intermediates of dexrazoxane, B and C appeared in the plasma at low levels upon completion of the dexrazoxane infusion and then rapidly decreased with half-lives of 0.6 and 2.5 hours, respectively. A plasma concentration of 10 μM of ADR-925 was also detected at the completion of the dexrazoxane intravenous infusion period, indicating, as these workers conclude that in this setting, dexrazoxane is rapidly metabolised.

### In humans, the terminal half-life of doxorubicin is up to 48 hours and that for dexrazoxane is approximately 2 hours

In humans, free doxorubicin has a distributive half-life of about 5 minutes and a terminal half-life of 20–48 hours, indicating fast uptake into tissues and slow elimination thereafter [18]. By contrast, after intravenous administration to patients with cancer plasma concentrations of dexrazoxane were observed to decline with an average half-life of 2.2 +/−0.42 hours [34].

In the phase I study by Earhart* et al* [35], following the administration of dexrazoxane as a 30-minute infusion, at the final time point of 15 hours (approximately seven dexrazoxane half-lives) concentration-time curves show that the mean plasma concentration of dexrazoxane is at the limit of detection. Given that dexrazoxane is <2% bound to plasma proteins [34], and that the uncharged parent compound rapidly equilibrates across the cell membranes [36], then intracellular concentrations will likewise be at or near zero. Moreover, since it is only the parent compound that is a catalytic inhibitor of Top2β [37–40], then undegraded Top2β protein remaining at 15 hours, together with newly synthesised Top2β protein, will represent an exclusive target for doxorubicin, which at 15 hours is present at a very substantial concentration within cardiac tissues [10–19].

### Partial suppression of Top2β protein at early times in cardiomyocytes

*Lyu et al* [4] treated H9C2 rat cardiomyocytes with 100-μM dexrazoxane for 0, 1, 2, 4 and 6 hours. Following incubation, cells were lysed and protein levels of Top2α and Top2β were determined by western blotting followed by detection using enhanced chemiluminescence using X-ray films or the Kodak Image Station 2000R. These visualisations show a time-dependent disappearance of Top2β, but with no observable effect upon the levels of Top2α. Yan* et al* [41] treated HTETOP cells *with* 100-μM dexrazoxane for 0, 3, 6, 12 and 24 hours; and in a parallel assay, cells were incubated for 24 hours with 1-, 5-, 25-, 100-, and 200-μM dexrazoxane. Following incubation, cells were lysed and protein levels of Top2α and Top2β were determined by western blotting with detection using enhanced chemiluminescence followed by densitometric analysis. The western blot images show that at all time points incubation with dexrazoxane resulted in partial suppression of Top2β protein levels. Paradoxically, at 24 hours, cells incubated with 200-μM dexrazoxane showed markedly less depletion of Top2β protein by comparison with cells exposed to concentrations of dexrazoxane within the range 5–100 μM for the same period. In summary, and repeating the concern expressed earlier, considerable caution must be exercised in extrapolating laboratory findings using cell preparations exposed continuously to high concentrations of dexrazoxane, to the clinical setting with rapidly declining concentrations of dexrazoxane.

### Suppression of Top2β protein levels is partial and transient

The western blot images presented by Deng* et al* [42] reveal mean Top2β protein expression of 47%, 32% and 64% in the hearts of C57BL (B6) mice that were sacrificed at 6, 24 and 48 hours, respectively, following the intraperitoneal administration of 200 mg kg^−1^ dexrazoxane, with the control group of mice receiving saline injections. According to Deng* et al,* the increased expression of Top2β protein observed at 48 hours indicates recovery from the depletion induced by dexrazoxane. In parallel experiments, mice were injected with 200 mg kg^−1^ dexrazoxane alone, 20 mg kg^−1^ doxorubicin alone or in combination whereby dexrazoxane was injected 30 minutes before doxorubicin; hearts were removed 24 hours later for western blot analysis. Relative to the control group, the mean Top2β protein expression was 63%, 95% and 73% in mice that received dexrazoxane, doxorubicin and a combination of both drugs, respectively. In reviewing their results, Deng* et al* conclude that the depletion of Top2β protein by dexrazoxane *in vivo* is ‘transient’. They speculate that transient depletion of Top2β protein by dexrazoxane reduces the risk or the duration of any adverse effects upon cardiomyocyte viability associated with loss of the Top2β enzyme.

### Rapid recovery of suppressed Top2β protein levels

The effect of dexrazoxane on Top2β protein levels was studied by Yeh [5] in mice following the intraperitoneal administration of 100 mg kg^−1^ dexrazoxane. Western blotting of harvested hearts shows that Top2β protein was ‘nearly undetectable’ [5] at 6 hours after the administration of dexrazoxane, but recovery of Top2β protein is visibly evident at the next time point of 12 hours.

### Degradation of Top2β protein levels is not unique to dexrazoxane

Preferential degradation of Top2β is a ubiquitous phenomenon that is observed using both catalytic inhibitors and Top2 poisons and is, by no means, unique to dexrazoxane. This much-observed effect has been reported in studies using VM-26 (teniposide), VM-16 (etoposide), ICRF-193, BNS-22, genistein, lycobetaine, bufalin and dexrazoxane, and in an array of cell types [4, 6, 7, 41–56, 116]. Despite the plethora of drugs that degrade Top2β protein, dexrazoxane remains the only product that is licenced for the prevention of cardiotoxicity associated with the use of anthracyclines; *a posteriori*, in isolation, this clinical fact provides presumptive evidence against any involvement of the Top2β protein in the cardioprotective mechanism of dexrazoxane.

### Top2β protein remains a target even at near-zero levels

Kersting* et al* [57] investigated if the expression of Top2α and Top2β enzymes in normal cells display variability between individuals and whether this variability affects the apoptotic response to the effects of doxorubicin. They tested their hypothesis using peripheral blood leucocytes, a class of cells that has been shown to undergo apoptosis in response to doxorubicin [58, 59]. Peripheral blood leucocytes were isolated from healthy volunteers and exposed to 1-μM doxorubicin *ex vivo* for 24 hours. Apoptotic cells were detected by staining with a fluorescent conjugate of Annexin V, a protein that has a high affinity for binding to the early apoptosis marker, translocated membrane phosphatidylserine. Total Top2 expression was measured as catalytic activity using an assay that measures the Top2-specific enzymatic conversion of highly catenated kDNA into nicked circular and relaxed circular DNA. Resolution of Top2 isoforms was undertaken using protein extracted from the nuclei of peripheral leucocytes followed by western blot analysis using Top2α and Top2β antibodies.

**Figure 2.**
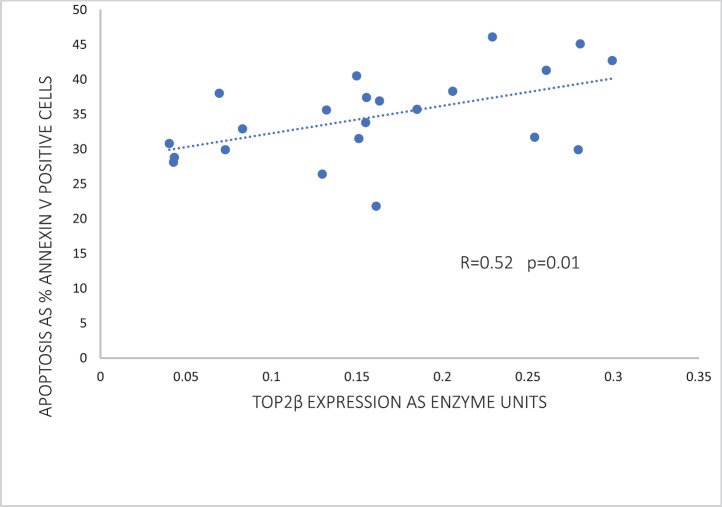
Correlation between Top2β expression and the percentage of apoptotic cells in peripheral blood leucocytes of 22 healthy volunteers following 24 hours ex vivo incubation with 1-μM doxorubicin. Data adapted from Kersting et al [57].

Of the two human Top2 proteins, only Top2β was detected by western blotting of the nuclear extracts of peripheral blood leucocytes. Strikingly, Top2β catalytic activity correlated significantly (*p* = 0.01) with the apoptotic response in peripheral blood leucocytes exposed to 1*-*μM doxorubicin (Figure 2). As discussed below, this correlation has a profound significance in refuting the argument that dexrazoxane exerts a cardioprotective effect by lowering Top2β protein levels within heart tissue.

However, before assessing the potential implications of this finding by Kersting* et al* [57], it is useful and necessary to explore the goodness-of-fit of these data. The increased variance above the median value of the abscissa (top right-hand corner) suggests that the correlation can be improved by weighting and exploring a nonlinear regression. Using the subscriber version of MyCurveFit, following extraction of the original data by digitisation, a weighted power curve regression yields *R* = 0.786 with *p* = 0.0003. Residual plot analysis (not shown here) confirms that the data of Kersting* et al* [57] can be represented adequately by both a linear regression and a weighted nonlinear regression. Notably, both regressions provide the same take-home message which is that at levels of Top2β protein expression close to zero (<0.05 as enzyme units) (Figure 2), there is clear evidence of substantial apoptosis (30%) following exposure to doxorubicin. Accepting these data, it is not unreasonable to conclude that if the cardioprotective effect of dexrazoxane is mediated by a dexrazoxane-induced lowering of Top2β protein levels then levels of Top2β protein need to be suppressed to near-zero levels and remain that way for a prolonged period (days and weeks), and with no provision for early recovery. However, as the animal and human data reviewed above indicate, that does not happen.

### Is the use of peripheral blood leucocytes relevant to the effects of doxorubicin within cardiac tissue?

Ten years after the seminal contribution by Kersting* et al* [57], Lipshultz* et al* [60] studied the mitochondrial DNA function in doxorubicin-treated childhood acute lymphoblastic leukaemia (ALL) survivors. From the outcomes of this long-term follow-up study, they cautiously propose that functional similarities may exist between the mitochondria of peripheral blood mononuclear cells (PBMCs) and those of cardiomyocytes and that the transcriptome of PBMCs might serve as a surrogate marker of doxorubicin-induced cardiotoxicity. Moreover, preliminary results by Yeh [5] are used in support of his proposal that Top2β protein levels of peripheral blood can be used to predict a patient’s susceptibility to anthracycline-induced cardiotoxicity [5].

Taken together, the author concludes that the earlier work by Kersting* et al* [57] is relevant to this analysis and the outcomes of that work permit the extrapolation that in the heart, near-zero levels of Top2β protein are a sufficient and functional target for doxorubicin.

In summary, there are no published data to show that in clinical practise dexrazoxane attenuates or compromises the cardiotoxicity of doxorubicin through any significant effects upon the levels of doxorubicin’s target, Top2β protein, and as presented next, new findings show that dexrazoxane is cardioprotective even when dexrazoxane-induced decreases in Top2β protein are blocked.

### New findings: Dexrazoxane is cardioprotective even when degradation of Top2β is blocked

Using their well-validated neonatal ventricular myocyte model, Hasinoff* et al* [61] investigated the cardiotoxicity of the proteasome inhibitors, bortezomib and carfilzomib alone and in combination with doxorubicin. While they show that bortezomib and carfilzomib were toxic to myocytes and induced apoptosis, they also observed that a brief pre-exposure of myocytes to nontoxic concentrations of bortezomib or carfilzomib markedly increased doxorubicin-mediated damage. Accordingly, they propose that in their model, the combination of doxorubicin with either bortezomib or carfilzomib may produce more than additive cardiotoxicity. Moreover, they add that these new results are worrying since the clinical use of doxorubicin, combined with either bortezomib or carfilzomib could potentially induce greater cardiotoxicity than the individual drugs used alone.

Importantly, the methodology of this study offers a unique opportunity to additionally investigate the mechanism of action of dexrazoxane.

With the use of proteasome inhibitors, Hasinoff* et al* [61] tested the ‘Top2β hypothesis’ by determining whether inhibition of dexrazoxane-induced proteasomal degradation of Top2β could block the ability of dexrazoxane to protect against doxorubicin-induced myocyte damage. That is, if dexrazoxane exerts its protective effects against doxorubicin-induced myocyte damage by inducing proteasomal Top2β degradation, then inhibition of the dexrazoxane-induced proteasomal degradation of Top2β through prior treatment with bortezomib or carfilzomib would be expected to eliminate or reduce the protective effects of dexrazoxane.

**Figure 3.**
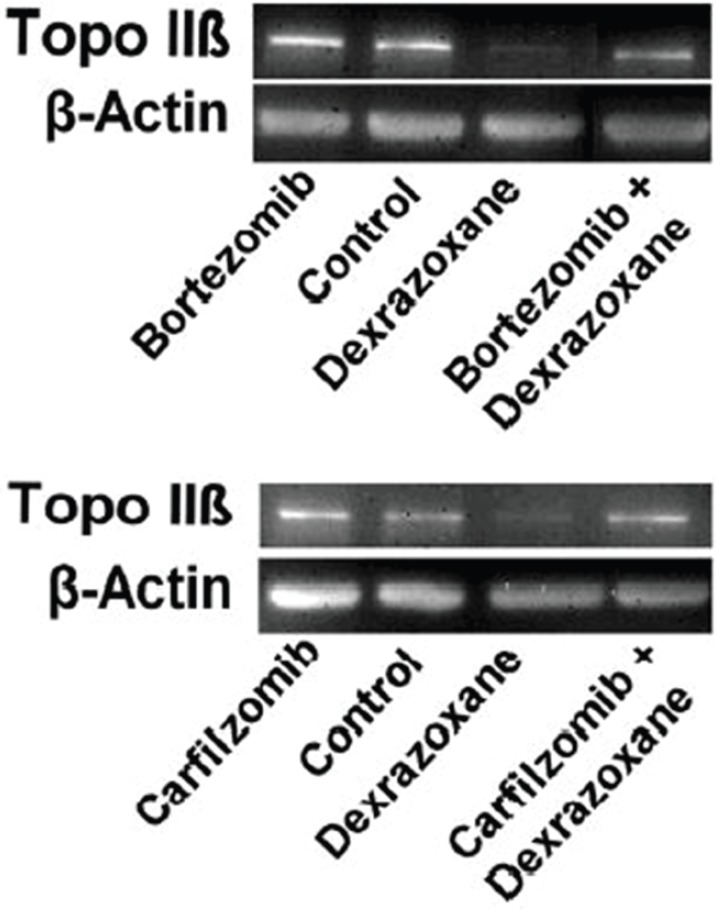
Effect of the proteasome inhibitors, bortezomib and carfilzomib on the dexrazoxane-induced decrease in Top2β levels in neonatal rat cardiomyocytes. Myocytes were treated with bortezomib (1 μM) or carfilzomib (2 μM) for 30 minutes in growth medium prior to a 6-hour treatment with dexrazoxane (100 μM), lysed and subject to sodium dodecyl sulphate polyacrylamide gel electrophoresis and western blotting. Images reproduced from Hasinoff et al [61] with permission (Springer; Copyright Clearance Licence: 4165441151593).

The effect of pre-treating myocytes with the proteasomal inhibitors, bortezomib or carfilzomib upon the dexrazoxane-induced reduction in Top2β protein levels are shown in Figure 3. Both inhibitors effectively prevented the dexrazoxane-induced proteasomal degradation of Top2β.

Having established that the proteasomal inhibitors effectively prevented the dexrazoxane-induced proteasomal degradation of Top2β, Hasinoff* et al* treated myocytes with a proteasomal inhibitor, a proteasomal inhibitor plus dexrazoxane, a proteasomal inhibitor plus doxorubicin or a proteasomal inhibitor plus doxorubicin and dexrazoxane. The damage was measured using the lactate dehydrogenase release assay, which is a widely used measure of drug-induced damage to cardiomyocytes [62, 63], and which has been validated as a sensitive and reliable assay for measuring anthracycline-induced injury and the protective effects of dexrazoxane upon neonatal rat ventricular cardiomyocytes [64].

As the outcomes from this elegant study show, despite the effective blockade of the dexrazoxane-induced proteasomal degradation of Top2β, the protective effects of dexrazoxane were largely maintained against the damaging effects of doxorubicin over a series of bortezomib and carfilzomib concentrations. Hasinoff* et al* conclude that *‘These results suggest that dexrazoxane does not protect against doxorubicin-mediated damage solely by reducing topoisomerase IIβ levels in the heart…*’

Moreover, they add that it is noteworthy that other workers using a rat model have also demonstrated that dexrazoxane prevents carfilzomib-induced cardiotoxicity, providing further support for a mechanism of action that is unrelated to a dexrazoxane-induced proteasomal degradation of Top2β [65, 66].

### Summary

Following a near-exhaustive survey of the literature, the evidence fails to support the assertion that the clinical cardioprotective effect of dexrazoxane is mediated by a dexrazoxane-induced lowering of the level of Top2β protein. At best, the *in vivo* results show that the lowering of Top2β protein level by dexrazoxane is transient and short-lived (hours) with a rapid recovery by newly synthesised Top2β protein. Within the context of persistently elevated levels of doxorubicin over a period of days and weeks, it is difficult to understand how in a clinical setting lowering of Top2β protein levels following a single 15-minute infusion of dexrazoxane, with a mean half-life of 2.2 hours [34], can translate into a cardioprotective effect.

Moreover, such a reductionist notion of Top2β protein lowering also fails to explain the long-term (years) cardioprotective effects of dexrazoxane [67] or the quantifiable differences in cell biology between long-term survivors who received doxorubicin by comparison with those who received doxorubicin plus dexrazoxane [60].

## Introducing the previously-unknown relationship between dexrazoxane and poly(ADP-ribose)

In 2014, the outcomes of a customised association rule learning algorithm (CEME…*Cutting Edge Medical Education*), powered to mine the published literature, reveal a previously-unknown relationship between dexrazoxane and poly(ADP-ribose) (PAR) polymer (methods described in [68]).

Historically, the construction of the CEME algorithm was pioneered by McCormack Pharma in the early 1980s from the fundamental tenet whereby a quasi-chaotic relationship exists between experimental data and real-world clinical outcomes. Restated, for a deterministic system that is chaotic, final outcomes are not predictable and are sensitive to starting values. However, in a quasi-chaotic context-dependent system, in common with behaviour in a chaotic system, small differences in starting values likewise result in large differences in final outcomes. However, unlike a true chaotic system, the relationship between the initial conditions and the final outcome is fixed, and accordingly, the experimental data uniquely code for a drug’s real-world clinical profile and patterns. In a quasi-chaotic system, CEME displays exquisite sensitivity in probing natural language text for the discovery of new and interesting associations that otherwise would remain lost.

The initial application in 2014 of CEME to dexrazoxane generated 43 outcomes from the published literature that when decoded, resulted in an association rule defining a previously unknown relationship whereby the mechanism of action of dexrazoxane involves some effect upon the synthesis of or an interaction with PAR polymer.

That is, dexrazoxane is a modulator, most likely an inhibitor of either the activity of the polymerase, PAR polymerase (PARP) (most likely PARP1) or PAR polymer function.

### About Poly(ADP-ribose)

**Figure 4.**
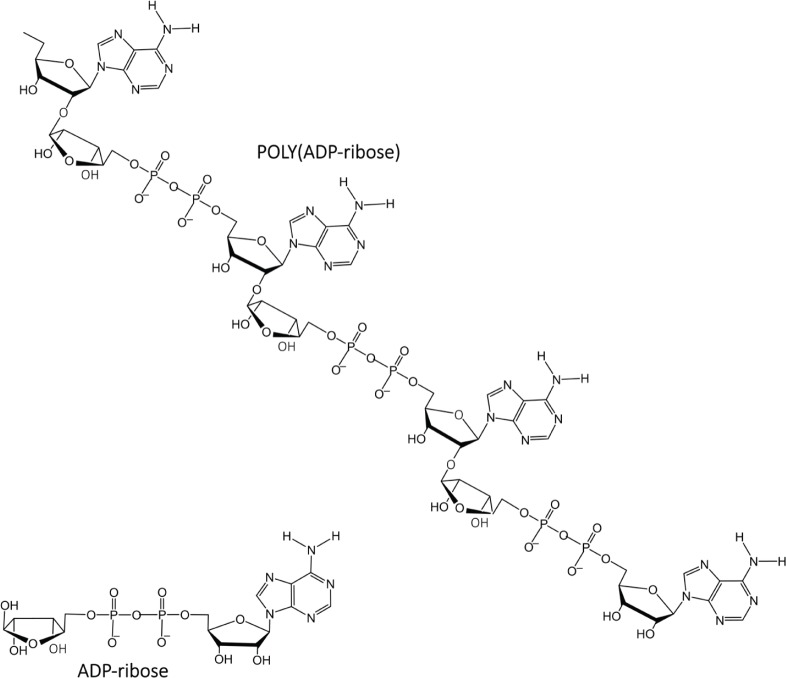
PAR is a polymer of ADP-ribose monomers.

PAR is a polymer of ADP-ribose monomers (Figure 4). PAR synthesis is tightly controlled in mammalian cells with steady-state levels being kept relatively low. However, DNA damage elicits immediate cellular responses that include activation of PARP enzymes with a resultant rapid increase in PAR levels [69]. Among the PARP family members, PARPs 1–3 are DNA damage responsive [70], and functioning as a DNA damage sensor, PARP1 is notable for its well-documented role in orchestrating complex and diverse mechanisms that result in either reparation of DNA lesions or the induction of cell death [71–73].

### PARP recognises damaged DNA

In regulating DNA topology, such as supercoils, the normal catalytic cycle of Top2β consists of a transient enzyme-bridged DNA DSB where the attachment of Top2β to the DNA is via a 5′ tyrosyl phosphodiester covalent linkage. Normally, these transiently cut ends enable relaxation of tangled DNA by a progressive restoration to a less chaotic and lower energy topology. Within the cardiomyocyte, doxorubicin, a Top2β poison targets Top2β and stabilises the Top2β-DNA cleavage complex upon the DNA with the result that the DSB is not religated. The effect of this doxorubicin-stabilised Top2β-DNA cleavage complex is to create a ‘transcriptional roadblock’ that arrests elongating RNA polymerase II in its progress to catalyse the transcription of DNA into messenger RNAs. The arrest of RNA polymerase II triggers the ubiquitin/26S proteasome-dependent degradation of Top2β. In clearing the stalled transcription machine, the DSB becomes exposed.

PARP1 is one of the first proteins to recognise damaged DNA. In the case of mild DNA damage, the cell activates PARP to facilitate DNA repair. When the levels of DNA damage are beyond the cellular repair capacity, programmed cell death is activated. Extensive DNA damage is accompanied by large-scale PAR polymer synthesis that may lead to a unique form of caspase-independent cell death, termed parthanatos (*Thanatos* from Greek Mythology) whereby PAR polymers translocate to the mitochondria and mediate the release of apoptosis-inducing factor (AIF) from the mitochondria. AIF then translocates to the nucleus and induces cell apoptosis by causing DNA fragmentation and chromatin condensation.

## Doxorubicin, PARP and cardiotoxicity

It has been shown that the activation of PARP contributes to the development of doxorubicin-induced heart failure, and PARP inhibitors have a protective effect [74–76].

Historically, Pacher* et al* [76] reasoned that activation of PARP may contribute to doxorubicin-induced cardiotoxicity. Using a dual approach of PARP1 suppression, by genetic deletion or pharmacological inhibition with the phenanthridinone-based PARP inhibitor PJ34, these workers demonstrated the role of PARP in the development of cardiac dysfunction induced by doxorubicin. PARP1+/+ and PARP1−/− mice received a single injection of doxorubicin. Five days after doxorubicin administration, left ventricular performance was significantly depressed in PARP1+/+ mice, but only to a smaller extent in PARP1−/− mice. Similar experiments were conducted in BALB/c mice treated with PJ34 or vehicle. Treatment with PJ34 significantly improved cardiac dysfunction and increased the survival of the animals. In addition, PJ34 significantly reduced the doxorubicin-induced increase in the serum lactate dehydrogenase and creatine kinase activities, but not metalloproteinase activation in the heart. From these observations, Pacher* et al* [76] conclude that PARP activation contributes to the cardiotoxicity of doxorubicin. They add that PARP inhibitors may exert protective effects against the development of severe cardiac complications associated with the use of doxorubicin.

Following this early work, the same group investigated the effect of a novel ultrapotent PARP inhibitor, INO-1001 on the development of heart failure induced by permanent ligation of the left anterior descending coronary artery, heart failure induced by doxorubicin and acute myocardial dysfunction induced by bacterial endotoxin [74]. In the coronary ligation model, a significantly depressed left ventricular performance and impaired vascular relaxation of aortic rings were found, and PARP inhibition significantly improved both cardiac function and vascular relaxation. In the doxorubicin model, a single injection of doxorubicin-induced high mortality and a significant decrease in left ventricular systolic pressure, stroke volume, stroke work, ejection fraction and cardiac output. Treatment with the PARP inhibitor reduced doxorubicin-induced mortality and markedly improved cardiac function. Importantly, PARP inhibition did not interfere with doxorubicin’s antitumor effect. In the endotoxin model of cardiac dysfunction, PARP inhibition attenuated the suppression of myocardial contractility elicited by endotoxin. Pacher* et al* [74] conclude that these new data strengthen their earlier view that PARP inhibition may represent an effective approach for the experimental therapy of various forms of acute and chronic heart failure, including that induced by doxorubicin.

Szenczi* et al* [75] investigated the effects of doxorubicin treatment upon intracellular calcium handling in the hearts of rats after 6 weeks of doxorubicin treatment, under baseline conditions, and in response to oxidative stress induced by hydrogen peroxide exposure *in vitro*. Following this, they investigated whether pharmacological inhibition of PARP with the PARP inhibitor, PJ34 affected the changes in myocardial mechanical performance and calcium handling in doxorubicin-treated hearts under normal conditions and in response to oxidative stress. Their results showed a marked elevation in intracellular calcium in the doxorubicin-treated hearts which was normalised by pharmacological inhibition of PARP. PARP inhibition also prevented the myocardial contractile disturbances and calcium overload that developed in response to hydrogen peroxide in the doxorubicin-treated hearts. They conclude that their results demonstrate that PARP activation contributes to the development of the disturbances in cellular calcium handling that develop in the myocardium in response to prolonged doxorubicin exposure, and that PARP inhibition beneficially affects chronic morphological and functional alterations in doxorubicin-affected hearts.

Cardiotoxicity studies were undertaken by Ali’s group at the School of Pharmacy and Pharmacology, University of Manchester using both male and female CD-1 mice. Consistent with the outcomes from the previous investigators, they observed that the clinically-active PARP inhibitor AG014699 ameliorates doxorubicin-induced cardiotoxicity [77]. They conclude that PARP inhibitors have a role in ameliorating the dose-limiting toxicity of doxorubicin.

## Does dexrazoxane target PARP or PAR?

**Figure 5.**
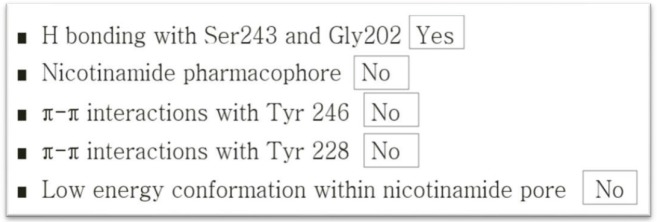
Lack of identity of dexrazoxane with a contemporary pharmacophore of PARP1.

Following the discovery by the author of a previously unknown relationship between dexrazoxane and PAR (for details of methods see [68]), it remained to determine whether dexrazoxane targets PARP or the polymer product, PAR. Initially, following scrutiny of literature reports of pharmacophore identification of compounds targeting PARP1 [78–85], the author concluded that dexrazoxane does not target PARP1. More recent publications [86–88] support this assertion. The overall lack of identity of dexrazoxane with a contemporary pharmacophore of PARP1 is summarised in Figure 5.

### In silico modelling reveals an interaction between dexrazoxane and PAR

**Figure 6.**
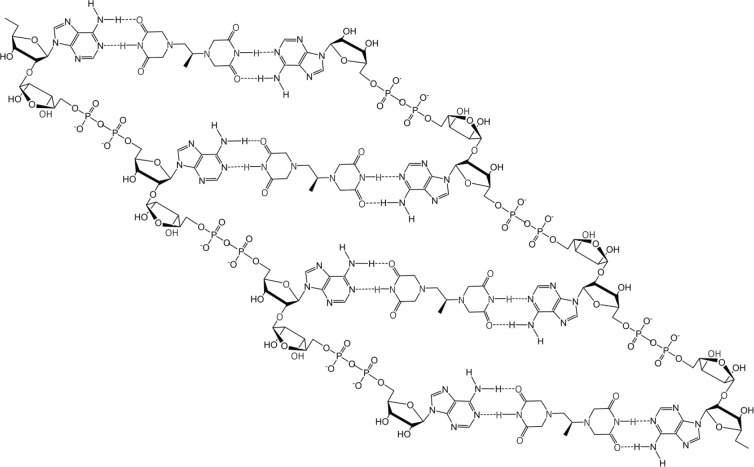
Dexrazoxane catalyses the formation of a hybrid self-assembled supramolecular structure between adjacent strands of PAR. This assembly depicts an antiparallel orientation of canonical Watson–Crick base pairing of dexrazoxane with adenine bases.

**Figure 7.**
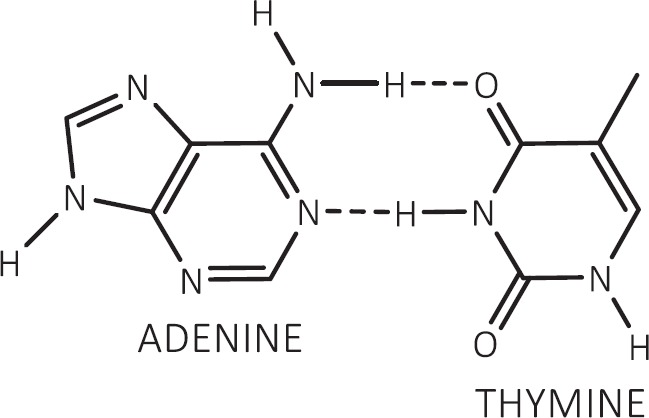
Canonical Watson–Crick adenine-thymine base pairing of double-stranded DNA.

If dexrazoxane is not an inhibitor of PARP, then it is not unreasonable to propose that dexrazoxane interacts with PAR. Strikingly, *in silico* modelling by the author shows that dexrazoxane has the capacity to catalyse the formation of a hybrid self-assembled supramolecular structure between adjacent strands of PAR. Graphically, this assembly depicts an antiparallel orientation of canonical Watson–Crick base pairing of dexrazoxane with adenine bases (Figure 6). That is, within this assembly the non-covalent attractive forces mimic the adenine-thymine base-pairing of double-stranded DNA (Figure 7).

### Horizontal forces stabilise the dexrazoxane-PAR self-assembly

**Figure 8.**
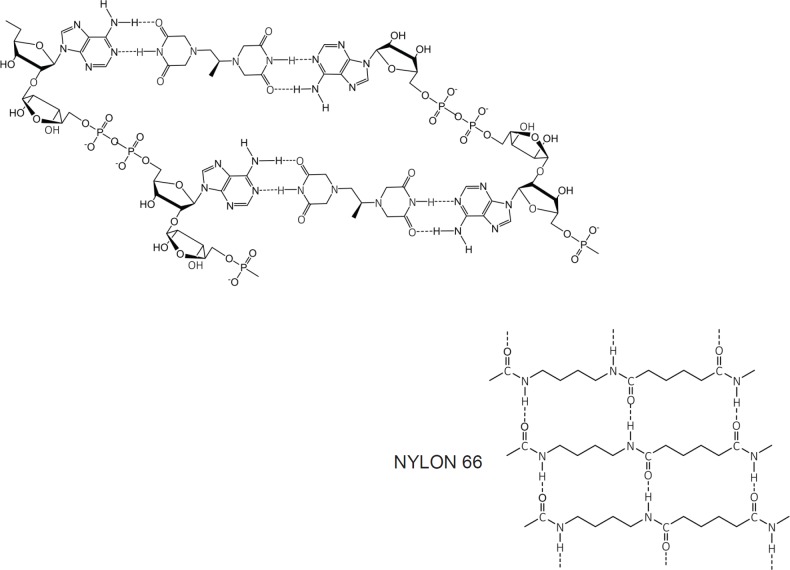
The dexrazoxane-catalysed supramolecular structure with PAR (top diagram) is characterised by two in-series hydrogen bonded base pairs at the level of each stack; this structural feature is reminiscent of the synthetic fibre, nylon 66 in which hydrogen bonds between adjacent polymer chains result in considerable tensile strength.

At first inspection, the dexrazoxane-catalysed supramolecular structure with PAR (Figure 6) is distinguished from a DNA duplex by two in-series hydrogen bonded base-pairs at the level of each stack that is directly attributable to the inclusion of dexrazoxane. Indeed, this novel feature is reminiscent of the synthetic fibre, nylon 66 in which hydrogen bonds between adjacent polymer chains result in considerable tensile strength (Figure 8).

The comparison between the dexrazoxane-catalysed supramolecular structure with PAR and the structure of the synthetic fibre, nylon 66 (Figure 8) is significant, not merely because both supramolecular assemblies demonstrate ‘in-series’ hydrogen bonding, but importantly because the stability of both assemblies is enhanced by an associated, cooperative phenomenon known as the ‘Gulliver Principle’ [89, 90]. In the same way that the fictitious giant, Gulliver in the land of Lilliput was bound to the ground by a great number of ropes, each of which could be easily broken, together, acting in concert they provided a formidable restraint.

From the outcomes of stability studies with DNA, the increased horizontal distance between PAR strands as a direct result of the interpolation by dexrazoxane (Figures 6 and 8) can be anticipated to significantly reduce the repulsive forces between adjacent hydrophilic phosphate groups and minimise destabilising effects of the phosphate-sugar backbone [91, 92].

Conformational studies by Minaga and Kun [93] of long-chain PAR in solution reveal that spectral shifts induced by divalent cations (Mg^2+^ and Ca^2+^) and the highly cationic polyamine, spermine are similar to that reported for DNA. From these seminal observations, Minaga and Kun [93] propose that in common with single-strand DNA, PAR can also adopt a helical conformation and that a double helix cannot be excluded. Importantly, stabilisation of a helical conformation of PAR by increased cationic pressure is entirely consistent with attenuation of the repulsive and destabilising effects of backbone phosphate anions as a direct result of the increased spacing between strands attributable to the inter-strand non-covalent incorporation of dexrazoxane.

**Figure 9.**
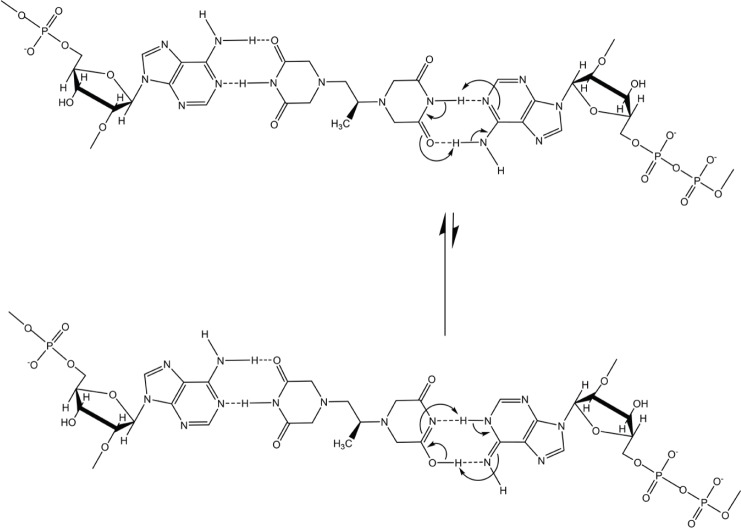
Tautomeric stabilisation of the dexrazoxane-adenine interaction.

In addition, the dexrazoxane-adenine interaction exhibits a dynamic stabilising equilibrium known as tautomerism that thermodynamically favours the keto-amine tautomer (Figure 9). Such mutually reinforcing hydrogen bonds are significantly more stable than regular hydrogen bonds.

By contrast with the horizontally orientated stabilising forces within the PAR-dexrazoxane supramolecular self-assembly, the DNA double helix is stabilised more by vertically orientated base stacking than by horizontal base pairing [91, 94–98]. This statement is consistent with the observation that free bases (adenine, thymine, cytosine and guanine) in water stack on top of one another rather than forming hydrogen-bonded pairs [95, 99–101]. Moreover, vertically orientated stacking interactions are unlikely to contribute to the stabilisation of the hybrid self-assembly of dexrazoxane with PAR given that the distance between the dexrazoxane-adenine stacked assemblies is considerably in excess of 3.4 Å (see below), which is the observed distance between stacked bases in DNA.

Using a Monte Carlo algorithm, Danilov* et al* [95] show that at the critical vertical separation of 3.4 Å, stacked base dimers (adenine-thymine; guanine-cytosine) are energetically favoured by comparison with the corresponding Watson–Crick hydrogen-bonded base pairs. Based upon the results of their simulations they propose that the preference for stacking is due to a significant interaction between each base and water. More recent computer simulations by Mak [91] show that the dominant driving force for stabilising base stacking within DNA is solvent entropy. That is, the simulations show that as the bases get closer to each there is a spontaneous release (‘squeezing out’) of ordered water molecules from the collective space that they occupy. Mak also demonstrates that stacking is not easily reversed and that at 3.4 Å, separation of stacked bases encounters an energy barrier. This barrier exists because released water cannot easily re-infiltrate the space now occupied by the stacked bases.

**Figure 10.**
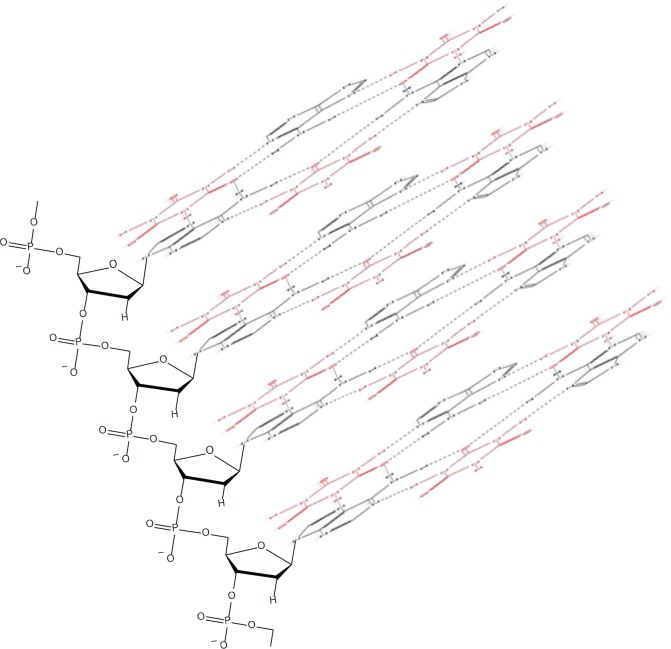
Cyanuric acid (red)-catalysed self-assembly of a poly(A) triplex. Adapted from Avakyan et al [102].

Such phenomena, however, cannot be predicted to occur within the PAR-dexrazoxane supramolecular self-assembly with a vertical separation between stacks (as horizontal planes) of approximately 10–12 Å. Interestingly, the assembly of the PAR analogue, polyadenine (poly(A)) with the thymine-containing dexrazoxane-like molecule, cyanuric acid likewise appears to depend upon horizontal forces for its robust stability, despite a vertical separation between the adenine-cyanuric acid-adenine stacks that is closer to 3.4 Å (Figure 10) [102, 103] (discussed in more detail below).

### Summary

Key features of the *in silico* modelling study of the interaction between dexrazoxane and PAR are summarised within Box 1.

Box 1. **Key features of the *in silico* modelling study of the interaction between dexrazoxane and PAR.**

- Each of the (bis)dioxopiperazine units of a single molecule of dexrazoxane has the capacity to simultaneously unite the adjacent strands of PAR by a non-covalent interaction with the adenine base of an ADP-ribose moiety of each strand.
- A stack of vertically orientated dexrazoxane molecules between adjacent strands of PAR can be accommodated within a theoretical thermodynamically stable low-energy supramolecular assembly.
- Each repeating subunit of this supramolecular assembly consists of one dexrazoxane molecule and an ADP-ribose moiety from each adjacent strand.
- Within each subunit the non-covalent attractive forces between dexrazoxane and PAR mimic the adenine-thymine base-pairing of double-stranded DNA.
- A thermodynamically stable self-assembly between dexrazoxane and PAR is consistent with a model that depicts an anti-parallel orientation of canonical Watson-Crick base-pairing of (bis)dioxopiperazine units with adenine bases.
- The dexrazoxane-catalyzed supramolecular assembly with PAR is distinguished from a DNA duplex by two in-series hydrogen-bonded base-pairs at the level of each stack (=subunit) that is directly attributable to the inclusion of dexrazoxane.
- Within supramolecular assemblies, repeating (stacked) in-series hydrogen bonds enhance tensile strength.
- By comparison with a DNA duplex, the increased horizontal distance between PAR strands due to interpolation by dexrazoxane reduces repulsive forces between adjacent hydrophilic phosphate groups that minimizes destabilizing effects of the phosphate-sugar backbone.
- The dexrazoxane-adenine interaction exhibits a dynamic stabilizing equilibrium (tautomerism) that thermodynamically favours the keto-amine tautomer; such mutually reinforcing hydrogen bonds are significantly more stable than regular hydrogen bonds.
- The findings herein also indicate that dexrazoxane has the capacity to self-assemble with PAR through a combination of both canonical and non-canonical Hoogsteen base-pairing resulting in the catalysis of a PAR triplex.
- Conformational analysis (see below) confirms that dexrazoxane can align with adenine bases of PAR and form both Watson Crick and Hoogsteen base pairs.

## The self-assembly of dexrazoxane with PAR accords with theoretical expectation

The discovery by the author that dexrazoxane self-assembles with PAR is not surprising. Previously, using an array of compounds, several workers have exploited the non-covalent interaction between adenine, the repeating base of PAR, and thymine (two thymine moieties (‘faces’) are integral within the (bis)dioxopiperazine moieties of dexrazoxane (Figure 1).

Mixing succinate-modified 2,4,6-triaminopyrimidine, an adenine structural analogue, with cyanuric acid, a thymine structural analogue (Figure 1), Cafferty* et al* [104] report the formation of extremely long supramolecular assemblies that retain water solubility. In common with the dexrazoxane-PAR assembly, it is the non-covalent interaction of the adenine structures with the thymine faces of cyanuric acid that drives the self-assembly [104].

**Figure 11.**
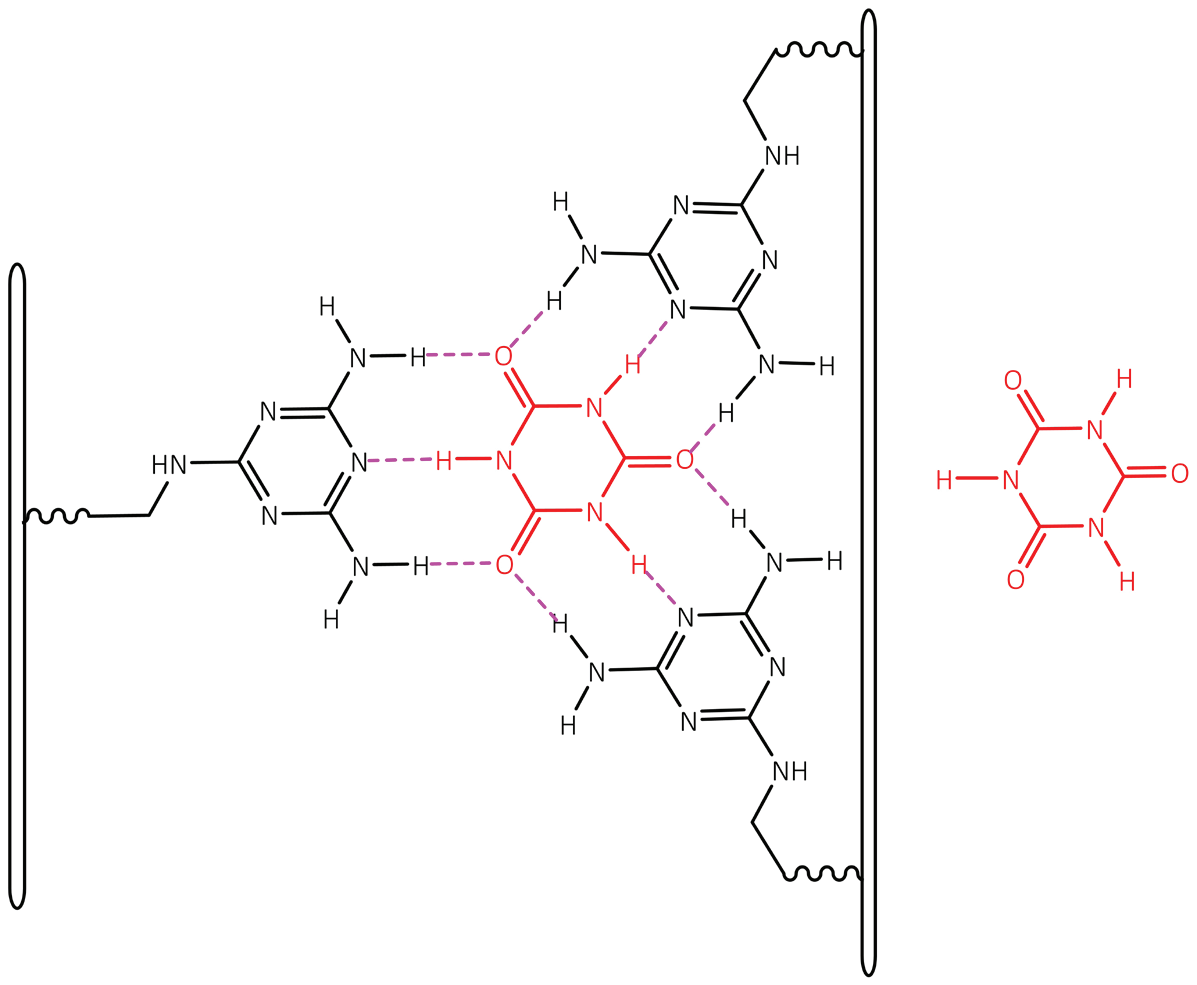
The three thymine faces of cyanuric acid (red) self-assemble with melamine-containing polyacrylate polymer strands. The hydrogen-bonding pattern shown is adapted from Zhou and Bong [105].

Of greater relevance to the interaction between dexrazoxane and PAR, however, is the study by Zhou and Bong [105] in which they used a model that incorporated repeating adenine-like structures within a polymer backbone. This study mimics the interaction of dexrazoxane with PAR, which, of course, is also a polymer with repeating adenine units. Inspired by Nowick* et al* [106, 107] who earlier had demonstrated stable adenine–thymine base pairing within aqueous sodium dodecyl sulphate micelles, Zhou and Bong derivatized a polyacrylate polymer strand that incorporated repeating units of the adenine structural analogue, melamine. Using this derivatized polymer, they observed that in an aqueous solution, cyanuric acid triggered the formation of a supramolecular self-assembly consisting of complementary non-covalent interactions between polyacrylate-anchored melamine units and monomeric cyanuric acid (Figure 11).

### A parallel model using a close analogue of PAR

**Figure 12.**
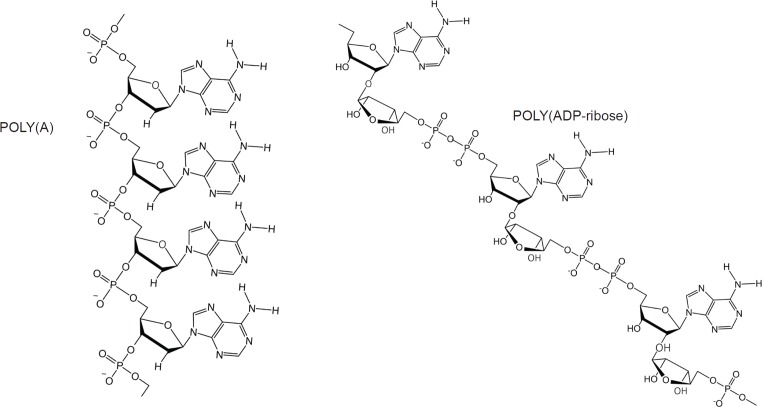
Poly(A) is closely related to PAR [poly(ADP-ribose)].

However, while these observations by Zhou and Bong [105] provide insights into the interaction between repeat adenine structures anchored to polymer strands and the thymine faces of a small molecule, it is, however, the more recent work of Sleiman* et al* [102] that is literally a facsimile of the author’s *in silico* modelling of the interaction between dexrazoxane and PAR. Also, using cyanuric acid, Sleiman* et al* observed supramolecular self-assemblies with a naturally occurring adenine-containing nucleotide, polyadenine (poly(A)), in both its DNA and RNA forms. Structurally, poly(A) is closely-related to PAR [poly(ADP-ribose)] (Figure 12), and as Figure 1 shows, dexrazoxane and cyanuric acid each contain thymine faces.

**Figure 13.**
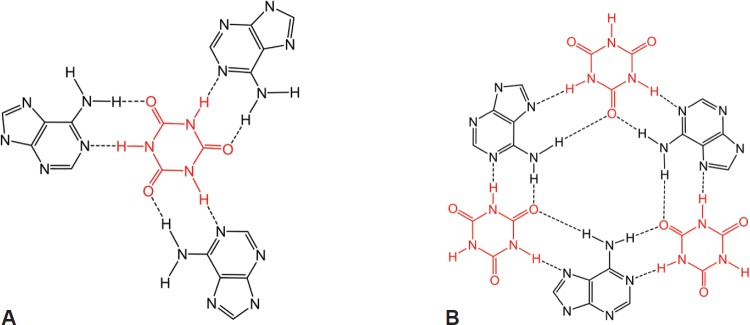
Cyanuric acid monomer(s) self-assembles with three poly(A) strands; canonical Watson–Crick base pairing is shown in (A) and a hexameric rosette structure formed by both canonical and non-canonical Hoogsteen base pairing is shown in B. Adapted from Avakyan *et al* [102] and Berger and Gazit [108]. (B) adapted with permission (Springer; Copyright Clearance Licence: 4165441151593).

Because cyanuric contains three ‘thymine faces’ (dexrazoxane contains two thymine faces), cyanuric acid was shown by Sleiman* et al* [102] to self-assemble with three poly(A) strands, as determined by circular dichroism and UV-visible spectroscopy. Cross-sectional plan views that depict the basic units of two different self-assemblies are shown in Figure 13A and B. Canonical Watson–Crick base pairing is shown in Figure 13A, and a self-assembly that combines both canonical and non-canonical Hoogsteen base pairing is shown in Figure 13B. Spectral studies by these workers suggest that the hexameric ‘rosette’ structure, depicted in Figure 13B, is the preferred assembly with cyanuric acid monomers uniting poly(A) strands as a coiling triplex formation. These workers also observed self-assembly using a synthetic adenine-containing peptide nucleic acid in place of poly(A).

A three-dimensional plan view in Figure 10, [for clarity, only a single strand of poly(A) is shown] depicts stacking of the assembly shown in Figure 13B, with the adenine bases (black) mapping the positions of the other two poly(A) strands in the triplex.

As an adjunct to the seminal publication by Sleiman* et al* [102], further discussion on this pioneering work using poly(A) and cyanuric acid is provided in the review by Berger and Gazit [108]. In addition, in recognising the contribution by Sleiman* et al* [102], the author wishes to acknowledge a later publication by Tateishi-Karimata and Sugimoto [103] in which these workers also demonstrate the utility of small hydrogen-bonding molecules in orchestrating the self-assembly of nucleic acids in water.

**Figure 14.**
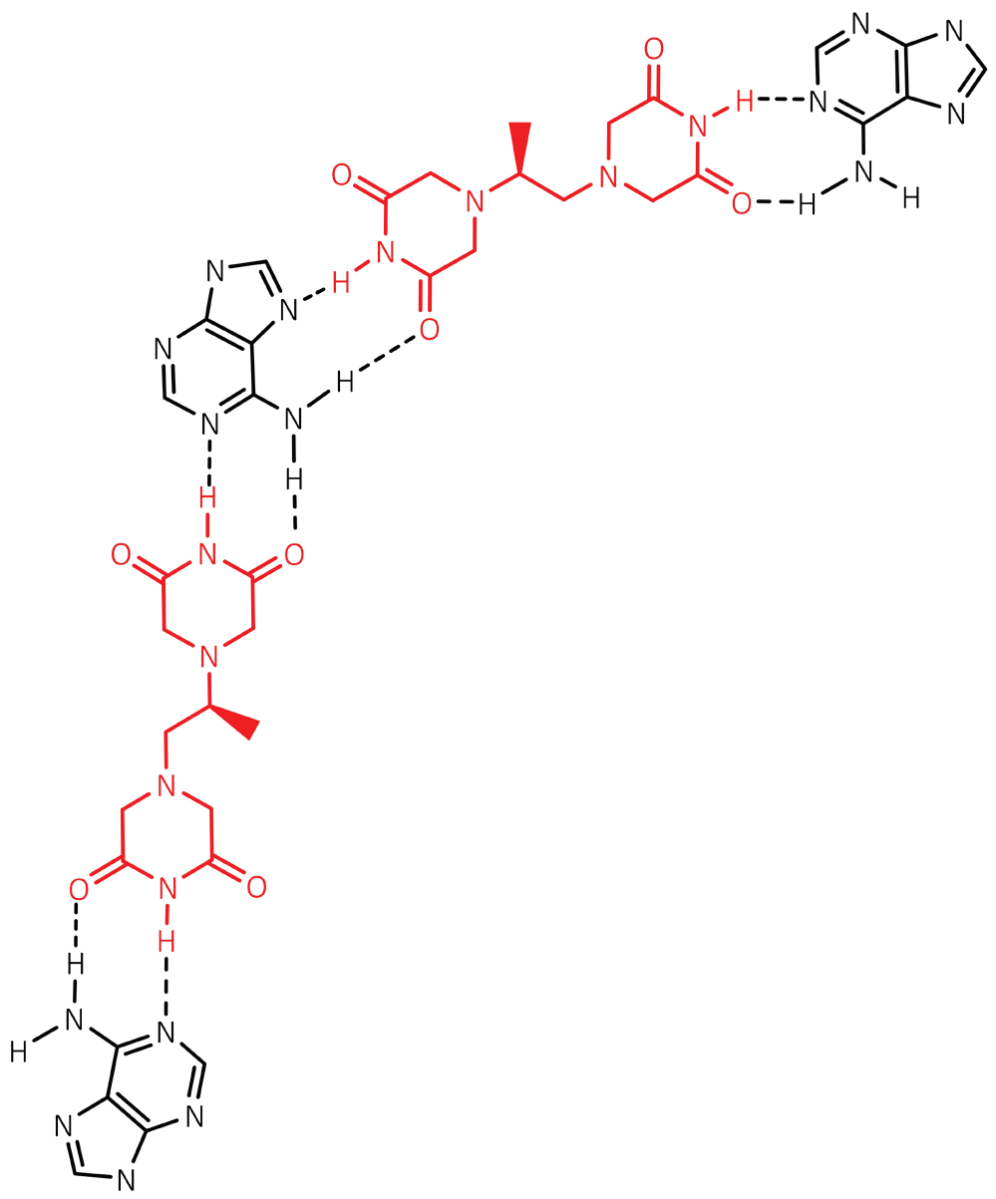
Dexrazoxane may also self-assembly with PAR through a combination of both canonical and non-canonical Hoogsteen base pairing to bring together a PAR triplex; the adenine moieties of PAR are shown in black. Furthermore, the contribution from both types of base pairing may lead to the formation of assemblies beyond a triplex.

Interestingly, this new report by Sleiman* et al* [102] provides a novel insight into the author’s own work that previously he had not considered, which is that in addition to the self-assembly of two PAR strands by canonical Watson–Crick base pairing (Figure 6), dexrazoxane may also self-assemble with PAR through a combination of both canonical and non-canonical Hoogsteen base pairing to bring together a PAR triplex, as suggested in the cross-sectional plan view in Figure 14. Moreover, the contribution from both type of base pairing may lead to the formation of assemblies beyond a triplex.

In concluding this section, it is both notable and instructive, that the discovery by McCormack Pharma, using an association rule learning algorithm, of an interaction between a nucleic acid and a ‘thymine-containing’ small molecule predates the publication by Sleiman* et al* by approximately 2 years [36]. Indeed, that breakthrough discovery by McCormack Pharma achieved initially by mining the existing data resources [36], provided the basis for a subsequent patent application (lapsed) by McCormack and George [109].

### Summary

Key features of published modelling studies of supramolecular self-assemblies using analogues of adenine and thymine or compounds with adenine-like or thymine-like groups in which non-covalent adenine-thymine base-pairing is mimicked are summarised within Box 2.

Box 2. **Key features of the published modelling studies of supramolecular self-assemblies using analogues of adenine and thymine or compounds with adenine-like or thymine-like groups in which non-covalent adenine-thymine base pairing is mimicked.**

- Historically, for an array of compounds, modelling of the adenine-thymine base-pair interaction has been supported by subsequent experimental observations of extensive supramolecular assemblies that are stable in both fully water-solvated systems and aqueous sodium dodecyl sulphate micelles.
- A model incorporating repeating adenine-like structures within a polymer backbone mimics the interaction of dexrazoxane with PAR, a polymer with repeating adenine bases. In aqueous solution, using derivatized polyacrylate polymer strands that incorporate repeating units of the adenine structural analogue melamine, the thymine structural analogue cyanuric acid [a thymine moiety is integral within the each of the (bis)dioxopiperazine units of dexrazoxane] triggered the formation of a supramolecular self-assembly consisting of non-covalent interactions between polyacrylate-anchored melamine units and monomeric cyanuric acid.
- Cyanuric acid catalyses the formation of supramolecular self-assemblies of a naturally occurring adenine-containing nucleotide, polyadenine (poly(A)), in both its DNA and RNA forms; structurally, poly(A) is closely related to PAR. Cyanuric acid-catalysed self-assemblies of poly(A) can be modelled using either canonical Watson–Crick base pairing or a combination of both canonical Watson–Crick and non-canonical Hoogsteen base pairing. Spectral studies suggest that the preferred assembly of poly(A) strands with cyanuric acid monomers is a coiling triplex formation consisting of both canonical and non-canonical base pairing.
- Cyanuric acid-catalysed self-assembly using a synthetic adenine-containing peptide nucleic acid (PNA) in place of poly(A) has also been demonstrated.

## Conformational analysis confirms that dexrazoxane aligns with adenine bases

Intuitively, it is to be expected that low-energy conformations will exist when there is a maximum separation between the piperazine structures. Conformational analysis undertaken by the author (for details of methods see the appended Supplement) confirm that lower energy conformations are consistent with the maximum spacing between piperazine structures with each adopting a half-chair conformation at methylene carbon atoms 3,5 of each ring structure resulting in an elevated tertiary amine nitrogen atom. As a half-chair conformation, the remainder of the piperazine structure is planar with the keto group at the carbon atom in position 2 of each ring structure in alignment with the tautomeric nitrogen of the secondary amine group of the adenine base of each ADP-ribose unit of PAR (using the orientation depicted in Figure 6). This analysis confirms that dexrazoxane can align with adenine bases of PAR and form both Watson–Crick and Hoogsteen base pairs.

## Self-assembly enables a deep compartment for the accumulation of dexrazoxane

**Figure 15.**
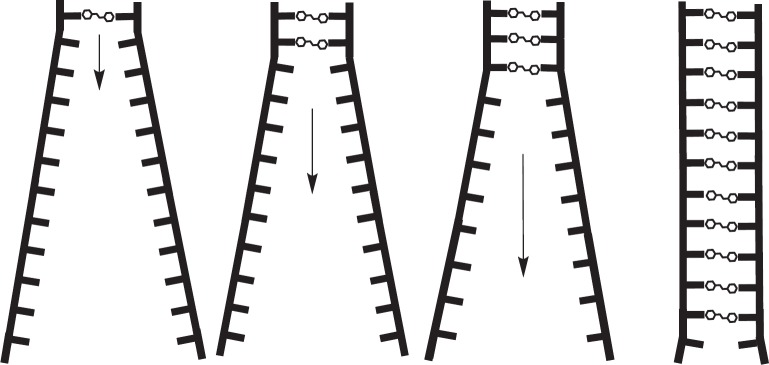
The anthracycline-compromised cardiomyocyte represents a deep compartment for the accumulation of dexrazoxane. Uncharged dexrazoxane transits the lipid membrane of the cardiomyocyte and encounters PARP-elaborated PAR within the intracellular compartment.

A model can be constructed that demonstrates the self-assembly process of dexrazoxane with PAR (Figure 15). For clarity, while Figure 15 depicts the self-assembly occurring at the termini of long-chain PAR strands (each strand can have a length in excess of 300 ADP-ribose units), dexrazoxane can interact with PAR at any part of the strand. Dynamically, the model illustrates how the anthracycline-compromised cardiomyocyte represents a deep compartment for the accumulation of dexrazoxane. Uncharged dexrazoxane transits the lipid membrane of the cardiomyocyte and encounters PARP-elaborated PAR within the intracellular compartment.

### Dexarazoxane selectively accumulates within doxorubicin-compromised cardiomyocytes

Dexrazoxane in assembly with PAR (Figure 15) is effectively trapped resulting in a concentration gradient of dexrazoxane into the intracellular compartment. Upon inspection, this model is clearly critically concentration sensitive. That is, in an *in vitro* simulation, high concentrations of dexrazoxane at early times above a critical concentration of dexrazoxane will saturate PAR strands and sterically hinder free molecules of dexrazoxane from binding simultaneously, but not necessarily coincidentally, with two strands of PAR. However, *in vivo,* it is likely that the concentration of PAR, especially at early times, is greatly in excess of the concentration of dexrazoxane with the result that free molecules of dexrazoxane can non-covalently bind with two strands of PAR. As discussed above (Figure 14), a combination of canonical and non-canonical Hoogsteen base-pairing may result in an interaction between dexrazoxane and PAR strands that is more complex than that illustrated in Figure 15.

## Dexrazoxane prevents AIF release from isolated mitochondria

**Figure 16.**
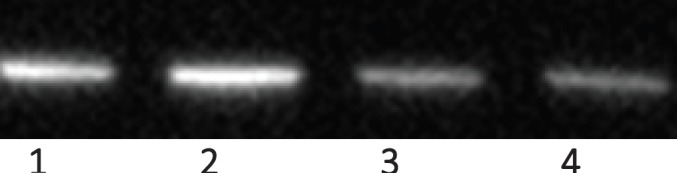
Dexrazoxane prevents AIF release from isolated mitochondria. Track 1 = Control; track 2 = 100 nM PAR and 100 nM dexrazoxane; track 3 = 100 nM PAR and 50 nM dexrazoxane; track 4 = 100 nM PAR and 10 nM dexrazoxane. PAR and dexrazoxane were incubated together for 10 minutes before adding to the mitochondrial suspension (western blots reproduced from the study final report).

In collaboration with Ritchie* et al* (Unpublished observations, Dr Kenneth J Ritchie, School of Pharmacy and Biomolecular Sciences, Liverpool John Moore’s University, Liverpool UK, Dec 2015), a pilot study was undertaken by the author to investigate the interaction between dexrazoxane and PAR. Using isolated rat liver mitochondria, we demonstrated for the first time that dexrazoxane inhibits PAR-induced release of AIF. Although the details of this methodology are not presented here, the western blots from this study, using anti-AIF antibody (Abcam ab32516) are reproduced in Figure 16.

Interestingly, and initially somewhat surprisingly, under the conditions of this preliminary study, we observed an inverse relationship between the concentration of dexrazoxane and the release of AIF from isolated mitochondria. With hindsight, this observation is consistent with a saturating concentration of dexrazoxane that within the short equilibrium period (10 minutes) binds extensively and randomly to individual PAR strands, thereby precluding organised and thermodynamically favoured self-assembly between dexrazoxane and PAR strands (Figure 15). Dose ranging and equilibrium times require further evaluation; notably, the parallel paradigm by Sleiman* et al* [102] using poly(A), a polymer closely related to PAR used an equilibration time of 12 hours.

Importantly, combining dexrazoxane with PAR *in vitro* is an inadequate paradigm of the *in vivo* deep compartment of the anthracycline-compromised cardiomyocyte described above. Further studies should incorporate the use of whole cells, such as isolated neonatal rat cardiomyocytes. The use of ionising radiation to induce DNA damage with the subsequent elaboration of PAR would enable a more exact study of the interaction between dexrazoxane and PAR with minimal confounding ([111], Personal communication Dr Kenny Ritchie Dec 2015). In this way, the lipid boundary imposed by the cell membrane will preclude saturation of PAR by dexrazoxane.

## Concluding remarks

Scientific truth is a moving target [112], and as the eminent Austrian Ethologist and Nobel Laureate, Konrad Lorenz famously remarked *‘Truth in science can be defined as the working hypothesis best suited to open the way to the next better one*’ [114].

Conceptually, all research consists of three basic components: *data*, *conclusions* and *hypotheses*. The not inconsiderable controversy about the truth of published research findings (claims) is elegantly argued by Ioannidis as being critically dependent upon the robustness of the conclusions [113]. The author of this review shares the view of Ioannidis [113], echoed by the remarks of Lorenz [114] that all too frequently most research conclusions are false. However, it is inescapable that in many scientific fields research claims will always include a component from prevailing bias. Consequently, that most conclusions are false is an inevitable part of the research endeavour and, accordingly, conclusions will always be subject to change; approaching the truth but never arriving. Accepting this (and I concede that many will not), it is the responsibility of scientific and clinical investigators to embrace uncertainty (and many do).

In this review, the author examines the burgeoning hypothesis that dexrazoxane protects the heart against anthracycline-induced damage by promoting the proteasomal degradation of Top2β, the Top2 isoenzyme that predominates in cardiomyocytes. This hypothesis has its origins within the guiding tenet that Top2β is required to initiate the entire phenotypic cascade of doxorubicin-induced cardiomyopathy. Consequently, it can be reasonably concluded that if doxorubicin is introduced into a system that is devoid of Top2β, then the effects of doxorubicin that are mediated by Top2β will not happen. This is a safe conclusion.

Several studies show that treatment with dexrazoxane results in the coincident depletion of Top2β protein. Taken together, we may conclude that the effects of both dexrazoxane and doxorubicin converge upon Top2β. This is a safe conclusion. Beyond this, no conclusions upon the relationship between Top2β and the cardioprotective mechanism of dexrazoxane are safe. Collectively, the above conclusions are ‘hypothesis generating’ and nothing more.

In the current examination of the ‘Top2β hypothesis’, at the outset, throughout and before arriving at any kind of conclusion, the author formulated key questions in an endeavour to reduce prevailing bias that otherwise might influence his own conclusions. Failing to do this and relying solely upon scrutiny and pooling of published ‘effect sizes’ will achieve little else other than reproducing the net bias that is inherent in the existing published claims. Such key questions include, but were not limited to:

- How many other compounds deplete levels of Top2β protein? Do compounds that deplete levels of Top2β protein protect the heart from the effects of anthracyclines? Indeed, it must be iterated that there is a consensus that the doxorubicin-stabilised Top2β-DNA cleavage complex creates a ‘transcriptional roadblock’ that arrests elongating RNA polymerase II; the arrest of RNA polymerase II triggers the ubiquitin/26S proteasome-dependent degradation of Top2β. In isolation, this observation raises alarm bells alerting us to the message that the depletion of Top2β protein is likely a ubiquitous phenomenon that has more to do with the proteasomal-mediated clearance of Top2β protein transcriptional roadblocks, and little if anything to do with the cardioprotective mechanism of a single drug.
- Is the cardioprotective efficiency of dexrazoxane compromised when the level of Top2β protein is manipulated (sustained), by blocking the ubiquitin/26S proteasome machine for example? The findings from the erudite study by Hasinoff* et al* [61] using dexrazoxane in combination with a proteasome inhibitor are provocative and demand a reappraisal of the earlier conclusions from other groups.
- What is known about the stoichiometry between doxorubicin, Top2β protein and cardiomyocyte toxicity? The seminal contributions by Kersting* et al* [57] and Lipshultz* et al* [60] permit the extrapolation by the author that in the heart near-zero levels of Top2β protein are a sufficient and functional target for doxorubicin. At best, the *in vivo* results show that the limited lowering of Top2β protein level by dexrazoxane is transient and short lived (hours) within a setting of a concomitant rapid recovery by newly synthesised Top2β protein.
- What is known about the relative mean residence times of the simultaneous, but not coincident administration of dexrazoxane and doxorubicin in clinical practise? Within the context of persistently elevated levels of doxorubicin over a period of days and weeks, it is difficult to understand how in a clinical setting, lowering of Top2β protein levels following a single 15-minute infusion of dexrazoxane with a mean half-life of 2.2 hours [34] can exert a cardioprotective effect.

### A different approach

In 2014, in the author’s hands, the outcomes of a customised association rule learning algorithm incorporating the use of antecedent surrogate variables (CEME, McCormack Pharma [68]) reveal a previously unknown relationship between PAR polymer.

Historically, CEME was conceived by the author’s Group in the early 1980s and has its origins in the fundamental tenet whereby a quasi-chaotic relationship exists between experimental data and real-world clinical outcomes.

Quasi-chaotic systems are characterised by a fixed relationship between initial conditions and the final outcome. Accordingly, experimental data uniquely code for a drug’s real-world clinical profile and patterns. In a quasi-chaotic system, CEME displays exquisite sensitivity in probing natural language text for the discovery of new and interesting associations that otherwise would remain lost.

The initial application in 2014 of CEME to dexrazoxane revealed an unequivocal signal defining a previously-unknown relationship between dexrazoxane and PAR polymer. Subsequently, the unique property of dexrazoxane to sequester PAR by base pairing was demonstrated using *in silico* modelling and a preliminary *in vitro* study confirmed an interaction. This previously-unknown relationship explains both acute and long-term cardioprotection in patients receiving anthracyclines. In addition, as an inhibitor of PAR dexrazoxane has access to the epigenome and this offers a new insight into protection by dexrazoxane against doxorubicin-induced late-onset damage [McCormack K, manuscript in preparation]. Notably, echocardiographic data suggest that dexrazoxane provides long-term cardioprotection, implying that prevention of cardiomyocyte damage during therapy can reduce the incidence of delayed doxorubicin-associated cardiomyopathy in long-term survivors without compromising the chances of oncological cure [117, 118].

What does the proposed association between dexrazoxane and PAR mean to Oncologists?

In addressing this important question some historical perspective must be introduced. Dexrazoxane reduces the incidence of anthracycline-induced heart failure by 80% and it is the only drug approved for its prevention [161]. Despite this impressive clinical effect, the use of dexrazoxane is somewhat limited largely because of historical concerns of interference with the antitumor efficacy of anthracyclines [157], induction of secondary malignancies and myelodysplastic syndrome [158]. However, subsequent meta-analyses do not support these concerns [159]. Moreover, prolonged survival of doxorubicin responders co-treated with dexrazoxane has been reported [160].

While beyond the scope of this review, the clinical consensus is that dexrazoxane does not compromise anti-tumour therapy. Indeed, in May 2017, the EMA Committee for Medicinal Products for Human Use approved the expansion of the Cardioxane Marketing Authorisation enabling increased use of dexrazoxane within the paediatric population for whom dexrazoxane had been previously contraindicated (dexrazoxane is marketed by Clinigen UK as a branded generic). A review of the evidence that historically led to a reassessment of the European Label for dexrazoxane found that dexrazoxane is not associated with an increased risk of second primary malignancies and that dexrazoxane therapy does not impair anthracycline’s anti-tumour efficacy [119].

Thus, if the above changes and revisions lead to an increase in the use of dexrazoxane throughout different patient populations, then the alert clinician should be responsive to novel/atypical/unexpected clinical benefits and outcomes if dexrazoxane is acting in some part by sequestering PAR by base-pairing.

Indeed, scrutiny of existing clinical outcomes supports an interaction between dexrazoxane and PAR. For example, Lipshultz* et al* propose that impaired cardiac function in doxorubicin-treated childhood cancer survivors is partly mediated by disruption of mitochondrial energy production and that this damage is abrogated by dexrazoxane [60]. In a long-term, cross-sectional study, these investigators examined mitochondrial DNA (mtDNA) copy numbers per cell and oxidative phosphorylation (OXPHOS) in PBMCs in 64 childhood survivors of high-risk ALL. Importantly, these workers argue that changes within PBMC mitochondria reflect what is occurring in cardiomyocyte mitochondria.

At a median follow-up of 7.8 years after treatment, doxorubicin-treated survivors without dexrazoxane had increased PBMC mtDNA copies per cell and concomitant use of dexrazoxane was associated with lower mtDNA copies per cell. The investigators add that mtDNA copies per cell in those who received dexrazoxane were within the normal ranges observed in their previous studies. OXPHOS activity was not different between groups. The investigators propose that in patients treated with doxorubicin alone, impaired mitochondria may undergo clonal expansion of mtDNA that functionally compensates for mutations or deletions that results in normal OXYPHOS and ATP production within the myocardium. They add that their findings support the evidence that dexrazoxane adjuvant therapy in paediatric high-risk ALL patients offers systemic mitochondrial protection and suggest a possible role of dexrazoxane prior to anthracycline therapy as a general protectant of mitochondrial function in other healthy tissues that include the ovaries [122].

Previously, Lebrecht* et al* [120] had proposed that mtDNA alterations initiated during acute doxorubicin exposure persist and accumulate during the long term and that this process may represent an important factor in the delayed onset of anthracycline-associated cardiomyopathy; they cite work that indicates some mtDNA mutations may persist for a long time because of inefficient repair [121].

Notwithstanding alternative explanations, can the above effects upon mtDNA be reconciled with an interaction between dexrazoxane and PAR, and in the absence of dexrazoxane can PAR explain the delayed onset of anthracycline-associated cardiomyopathy?

Using wild-type and PARP1-depleted A549 cells, Szczesny* et al* [123] investigated the role of PARP1 in the repair of mtDNA under oxidative stress conditions. They showed that intra-mitochondrial PARP1 interacts with mitochondrial-specific DNA base excision repair enzymes, endonuclease G-like 1 (EXOG) and DNA polymerase gamma (Polγ), which under oxidative stress become PARylated (a post-translational modification whereby PAR polymers are covalently-attached to proteins by PARPs). Notably, they observed that the presence of PARP1 adversely affected the integrity of mtDNA which was attributable to a pronounced inhibition of the repair of mtDNA. By contrast, repair of oxidative-induced damage to the mitochondrial DNA in PARP1-depleted cells was found to be more robust, and by comparison with the wild-type cells, this was associated with an enhancement of mitochondrial biogenesis. Szczesny* et al* speculate that by contrast with the well-established positive regulatory role of PARP1 in maintaining nuclear DNA integrity, the addition of PAR groups on mtDNA repair enzymes may uniquely reduce their affinity to bind with DNA and thus may attenuate the rate of mtDNA repair.

Accepting the new hypothesis that dexrazoxane sequesters PAR by base pairing, then arguably this sequestration state is comparable/equivalent to the PARP1-depleted cells used by Szczesny* et al* [123] and that sequestration of PAR by dexrazoxane can be envisaged to compromise the complex PARylation process [124]. For example, sequestration of PAR by dexrazoxane may disrupt the alignment of the highly negatively charged backbone of PAR polymers and accordingly disrupt electrostatic interactions between the PARylated substrate and proteins. In addition, sequestration of PAR may influence recognition of the PARylated substrate by so-called ‘ADP-ribose readers’ that contain ADP-ribose/PAR-binding domains [124]. Accordingly, the abrogation of impaired mitochondrial function by dexrazoxane reported by Lipshultz* et al* [60] may be attributable in part to an attenuation by dexrazoxane of the potential inhibitory effects of PARylation upon mtDNA repair following exposure to anthracycline therapy [120].

A further example serves to illustrate how the existing data can be re-evaluated in the light of an interaction between dexrazoxane and PAR. While the acute cardiotoxic effects of doxorubicin are consistent with a doxorubicin-induced DNA damage response resulting in both PARP1-dependent apoptotic and necrotic cell death [125, 126], delayed-onset doxorubicin-induced cardiomyopathy that may become manifest years or decades following the initial administration of doxorubicin, in addition to protracted effects upon the integrity of mtDNA [60, 120, 123] is also consistent with epigenetic alterations that induce a pathological shift in the relative expression of myosin heavy chain (MHC) isoforms [135, 152].

Two isoforms of MHC are expressed in the mammalian heart, α-MHC and β-MHC. The α-MHC isoform has a higher ATPase activity than β-MHC, and the contractile velocity of the heart is correlated with the relative amount of each isoform; hearts expressing greater amounts of α-MHC having a more rapid contractile velocity [136–138].

The α- and β-MHC ratio correlates directly with the overall cardiac performance in both animals and in patients with cardiomyopathy and heart failure [139–145]. Pathological hypertrophy of adult hearts is associated with α-MHC downregulation and β-MHC induction, which is a relationship that characterises the foetal phenotype of MHC expression [146]. However, hearts expressing α-MHC have better outcomes under stress conditions than those expressing mainly β-MHC [139, 140, 142]. Consequently, strategies that prevent or reverse undesirable shifts in the relative proportions of MHC isoforms represent attractive and fundamental approaches in the management of cardiomyopathies [147].

De Beer’s Group in the Department of Medical Physiology at Utrecht University observed that long-term administration of doxorubicin resulted in an impairment of the actin-myosin interaction in the hearts of male Wistar rats [148]. Notably, assays of ventricular muscle revealed a shift from the adult expressing high-ATPase isoform, α-MHC, to the foetal low-ATPase isoform, β-MHC. From these results and that of an earlier study by the same group [149], these workers conclude that the direct effect of chronic treatment with doxorubicin upon the actin–myosin system provides an additional mechanism through which anthracyclines exert their cardiotoxic effects.

Following their earlier observations of the effects of chronic treatment with doxorubicin upon the contractile function of the heart, de Beer* et al* investigated whether the cardio-protective properties of dexrazoxane involve the prevention of the deleterious effects of doxorubicin upon the actin–myosin contractile machinery [150]. They observed that dexrazoxane treatment offered significant protection against the doxorubicin-induced impairment of kinetics; time constants describing transitions between different states in the actin–myosin interaction were restored to normal when trabeculae were exposed to sudden changes in length in the so-called ‘quick release’ and ‘slack-test’. Tissue from both right and left ventricles was examined for MHC content using gel electrophoresis, and laser scanning densitometry was used to identify differences in myosin isoform composition. Whereas doxorubicin treatment significantly increased the β-MHC to α-MHC ratio, notably, dexrazoxane prevented the shift from the high ATPase α-MHC isoform towards the low ATPase β-MHC isoform.

Interestingly, there are no reports within the literature of an association between Top2β and MHC isoforms, and additional studies by de Beer* et al* do not support a role for free radicals in mediating the deleterious effects of doxorubicin upon the actin–mysoin contractile machinery [150]. However, delayed-onset toxicity associated with exposure to doxorubicin, may be explained by a phenotypic switch that results from doxorubicin-induced alteration(s) at the epigenome [McCormack K, manuscript in preparation]. Recently, Ferreira and coworkers using male Wistar rats, demonstrated that doxorubicin modulates gene expression patterns via alterations to the epigenetic landscape, notably the DNA methylome in the heart but not liver and that such changes contribute to doxorubicin-induced delayed-onset cardiomyopathy [151]. Moreover, disrupted cardiac mitochondrial biogenesis, as demonstrated by decreased mtDNA levels (see above) was also observed.

Are doxorubicin-induced alterations at the epigenome as manifest by a shift in the ratio between MHC isoforms and the effects of dexrazoxane in abrogating this shift, consistent with an interaction between dexrazoxane and PAR?

Hang* et al* in the Division of Cardiovascular Medicine at Stanford University School of Medicine show that Brahma-related gene 1 (Brg1), a chromatin-remodelling ATPase protein subunit of the BRG-1-Associated Factor (BAF complex), interacts with two other classes of chromatin-modifying enzymes, histone deacetylase (HDAC) and PARP, to regulate gene expression during cardiac growth, differentiation and hypertrophy [152]. In adults, Brg1 is turned off in cardiomyocytes. However, it is re-activated by cardiac stresses and Brg1 complexes with its embryonic partners, HDAC and PARP, to induce a pathological shift from α-MHC to β-MHC expression. In addition to HDACs, PARP1 is the only other chromatin-modifying enzyme known to regulate cardiac hypertrophy [153–155]. In addition, Hang* et al* show that in hypertrophic hearts PARP1 is bound to the proximal promoters of *α-MHC* and *β-MHC*, and that inhibition of PARP1 activity by the phenanthridinone-based PARP inhibitor PJ34 reduced both Brg1-mediated *α-MHC* repression and *β-MHC* activation in reporter assays, indicating that Brg1 and PARP1 cooperate to regulate *MHC* expression [152].

Notably, *Mahesh Gupta’s Group in the Department of Cardiothoracic Surgery at The University of Chicago* demonstrate that nuclear extracts treated with anti-ADP-ribose antibodies reveal increased poly-ADP-ribosylation (PARylation) of nuclear proteins in failing hearts by comparison with controls, thus confirming the presence of PAR polymer [156]. From this observation, they propose that post-translational modification of proteins by PAR interferes with their ability to bind to each other and to DNA, resulting in repression of gene transcription and finally cell death. Attenuation of cardiac hypertrophy in PARP^−/−^ mice was also evident from the analysis of hypertrophic marker genes. In PARP^+/+^ aortic artery-banded mice, the levels of β-MHC mRNA were highly elevated, whereas α-MHC levels were repressed, as expected. By contrast, in PARP^−/−^ aortic artery-banded mice, no repression of α-MHC levels was observed, and the levels of β-MHC were increased to a much lesser extent.

In their concluding remarks, they propose that PARP inhibitors may be useful agents for managing cardiomyopathies and heart failure.

Taken together, these and other data are provocative and indicate that in some part, the cardiotoxic effects of doxorubicin and the cardioprotective effects of dexrazoxane, converge upon the epigenome. By comparison with acute damage, delayed-onset toxicity is consistent with a phenotypic switch that results from doxorubicin alteration(s) at the epigenome. Sequestration of PAR through both canonical Watson–Crick base pairing, and non-canonical Hoogsteen base-pairing provides a mechanism for the cardioprotective effects of dexrazoxane against the toxic effects of doxorubicin in the acute term, and abrogation of delayed-onset cardiomyopathy.

Given the focal role of PAR in an overwhelming number of diseases, it is indeed thought provoking that dexrazoxane offers, for the first time, a unique opportunity to investigate the impact of sequestration of PAR, by comparison with inhibition of PARP, within a remarkably broad context. By way of example, and whilst beyond the scope of this review, the interaction between dexrazoxane and PAR offers the prospect that dexrazoxane can function as a cytoprotectant within other physiological systems (Lipshultz* et al* have previously suggested a possible role of dexrazoxane prior to anthracycline therapy as a general protectant of mitochondrial function in healthy tissues in addition to the heart [60]). Neuroprotection offered by dexrazoxane in a preclinical model of neurological dysfunction outperformed that of established neuroprotectants that included a blocker of the N-methyl-D-aspartate receptor channel, dizolcipline (MK-801), a calcium channel blocker, nimodipine, and an iron chelator, deferoxamine (desferal), resulting in significantly greater survival rates and attenuated neurological deficits [115]. In that study, Rodriguez* et al* [115] conclude that dexrazoxane has ‘*very important neuroprotective properties*’*.*

### Limitations

The CEME algorithm is a well-validated methodology that has been successfully applied by McCormack Pharma to many drugs throughout more than three decades [68, 127–134, 187]. CEME is a process that discovers ‘*previously unknown relationships*’ within the published literature for a nominated drug within an elected therapeutic category. By comparison with the kind of data stored in structured databases, natural language text within the published literature is unstructured, amorphous and difficult to mine using the traditional algorithms. In practise, the greatest obstacle is missing data within a dataset that is small (thousands of data pieces) by comparison with the much larger size (millions of data pieces) of dataset sets mined using methods traditionally applied in commercial operations such as mining high street supermarket transactions. CEME overcomes the problem of missing data by adapting the use of a statistical method known as ‘*antecedent surrogate variables*’*.* Specifically, in exploring the mechanism of action of dexrazoxane as a cardioprotectant, the task was to characterise as exhaustively as possible cell signalling within all known molecular phenotypes (as determined by extracellular first messengers) of anthracycline-exposed cardiomyocytes. In practise, this is an extremely demanding operation that requires the manual input for all known molecular phenotypes of all known second messengers, enzymes, receptors, transcription factors and so on. To reduce the possibility of a type I error (false positive), in addition to dexrazoxane, the CEME process should be repeated for all known cardioprotectants of anthracycline-exposed cardiomyoctes; the list includes beta blockers, angiotensin inhibitors, angiotensin-converting enzyme inhibitors and statins. That is, previously unknown relationships between each cardioprotectant and cell signalling within the anthracycline-compromised cardiomyocyte should be explored. For practical reasons, this was not undertaken.

*In silico* modelling studies demonstrate that dexrazoxane catalyses the formation of a hybrid self-assembled supramolecular structure between adjacent strands of PAR. These assemblies depict an antiparallel orientation of canonical Watson–Crick base pairing of dexrazoxane with adenine bases. In addition, these *in silico* studies show that dexrazoxane can also base pair with PAR strands by non-canonical Hoogsteen base pairing. Importantly, these supramolecular structures employing either canonical or non-canonical base pairing and combinations of the two, accord with the theoretical expectation of low-energy thermodynamically stable entities. Moreover, that such supramolecular assemblies are possible is strongly suggested by the work of other groups using molecules that are closely analogous to both dexrazoxane and PAR [102, 104–107]. However, confirmation that the supramolecular assemblies between dexrazoxane and PAR are stable under *in vivo* conditions is lacking. Initially, aqueous stability could be explored by utilising *in vitro* methods such as thermal denaturation using circular dichroism and ultraviolet–visible spectroscopy [102]. These critically necessary studies remain to be undertaken.

The results of a preliminary *in vitro* study show that consistent with expectation, dexrazoxane prevents PAR-induced AIF release from isolated mitochondria. However, dose-ranging studies are needed, and equilibrium times require investigation. The utilisation of exogenous PAR as used by the author is not a paradigm of a deep compartment for the accumulation of dexrazoxane and consequently does not adequately represent the *in vivo* situation. Preferably, further studies should incorporate the use of whole cells, such as isolated neonatal rat cardiomyocytes together with the use of ionising radiation to induce DNA damage with subsequent elaboration of endogenous PAR.

## Supplementary materials and methods

The conformational relationship of the non-covalent interaction between dexrazoxane and PAR was explored using a modified version of Allinger’s Molecular Mechanics MM2 force field (for review see [110]). Accordingly, conformational analysis with a dihedral driver was performed and minimum energy conformations were computed for dexrazoxane using Chem3D 16.0 with a minimum root mean squared gradient set to 0.1. Semi-empirical molecular orbital calculations were also undertaken using the Chem3D 16.0 tool MOPAC in order to eliminate unstable high energy conformers and for geometry optimisation. Conformational analysis and energy minimisation was additionally supported by the Chem3D 16.0 tool, CONFLEX (CONFLEX Corporation). Dissociation constants as pKa that were not evident within the literature were calculated using methods provided within Chemaxon (MarvinSketch).
